# Epigenome-wide analysis of maternal exposure to green space during gestation and cord blood DNA methylation in the ENVIR*ON*AGE cohort

**DOI:** 10.1016/j.envres.2022.114828

**Published:** 2023-01-01

**Authors:** Rossella Alfano, Esmée Bijnens, Sabine A S Langie, Tim S Nawrot, Brigitte Reimann, Kenneth Vanbrabant, Congrong Wang, Michelle Plusquin

**Affiliations:** a Centre for Environmental Sciences, Hasselt University, Agoralaan Building D, 3590, Diepenbeek, Belgium; b Department of Pharmacology & Toxicology, School for Nutrition and Translational Research in Metabolism (NUTRIM), Maastricht University, Maastricht, the Netherlands; c Department of Public Health, Leuven University (KU Leuven), Leuven, Belgium

**Keywords:** Maternal green space, Epigenomics, DNA methylation, Epigenome-wide, Differentially methylated regions, Gene expression, 450K, Infinium HumanMethylation450 BeadChip, *A1BG-AS1*, A1BG antisense RNA 1 lnRNA 887, β, beta-value, BH, Benjamini–Hochberg, *COL11A1*, the collagen type XI alpha 1 chain, DMP, differentially methylated position, DMR, differentially methylated region, *DUSP22*, dual specificity phosphatase 22, ENVIR*ON*AGE, ENVIRonmental influence ON early AGEing, EPIC, Illumina HumanMethylationEPIC Bead-Chip array, EWAS, epigenome-wide association study, FDR, false discovery rate, GWAS, genome-wide association study, *HTR2A*, 5-hydroxytryptamine receptor 2A, *HLA-DRB5*, major histocompatibility complex, class II, DR beta 5, HPA, hypothalamic-pituitary-adrenal, I_2_, heterogeneity statistic, IVW, inverse-variance weighted, lncRNAs, long non coding RNAs, *KCNQ1DN*, KCNQ1 downstream neighbour, M, methylated allele, mPRε, membrane progesterone receptor ε, NDVI, normalized difference vegetation index, *PAQR9*, class II progestin and adipoQ receptor 9, *PHF17*, PHD finger protein 1, PM_2.5_, particulate matter with an aerodynamic diameter <2.5 μg/m³, *PRSS36*, serine protease 36, r, Pearson's correlation, *RPTOR*, regulatory associated protein of mTOR complex 1, *SLC38A4*, the solute carrier family 38 *member* 4, *SPON2*, the spondin, U, unmethylated allele, *ZNF27*4, zinc finger protein 274

## Abstract

**Background:**

DNA methylation programming is sensitive to prenatal life environmental influences, but the impact of maternal exposure to green space on newborns DNA methylation has not been studied yet.

**Methods:**

We conducted a meta-epigenome-wide association study (EWAS) of maternal exposure to green space during gestation with cord blood DNA methylation in two subsets of the ENVIR*ON*AGE cohort (N = 538). Cord blood DNA methylation was measured by Illumina HumanMethylation 450K in one subset (N = 189) and EPICarray in another (N = 349). High (vegetation height>3 m (m)), low (vegetation height<3 m) and total (including both) high-resolution green space exposures during pregnancy were estimated within 100 m and 1000 m distance around maternal residence. In each subset, we sought cytosine-phosphate-guanine (CpG) sites via linear mixed models adjusted on newborns' sex, ethnicity, gestational age, season at delivery, sampling day, maternal parity, age, smoking, education, and estimated blood cell proportions. EWASs results were meta-analysed via fixed-effects meta-analyses. Differentially methylated regions (DMRs) were identified via ENmix-combp and DMRcate algorithms. Sensitivity analyses were additionally adjusted on PM_2.5_, distance to major roads, urbanicity and neighborhood income. In the 450K subset, cord blood expression of differentially methylated genes was measured by Agilent microarrays and associated with green space.

**Results:**

147 DMRs were identified, 85 of which were still significant upon adjustment for PM_2.5_, distance to major roads, urbanicity and neighborhood income, including *HLA-DRB5, RPTOR, KCNQ1DN, A1BG-AS1, HTR2A, ZNF27*4, *COL11A1* and *PRSS36* DMRs. One CpG reached genome-wide significance, while 54 CpGs were suggestive significant (p-values<1e-05). Among them, a CpG, hypermethylated with 100 m buffer total green space, was annotated to *PAQR9,* whose expression decreased with 1000 m buffer low green space (p-value = 1.45e-05).

**Conclusions:**

Our results demonstrate that maternal exposure to green space during pregnancy is associated with cord blood DNA methylation, mainly at loci organized in regions, in genes playing important roles in neurological development (e.g.*, HTR2A*).

## Highlights

• The impact of maternal exposure to green space on newborns DNA methylation has not been studied yet.
• We lead the first EWAS of green space in pregnancy and cord blood DNA methylation.
• 147 DMRs were found differentially methylated in the meta-EWASs.
• 85 DMRs were significant after adjustment for gestational exposure to air pollution.
• A DMP (p < 1-e05) was located on *PAQR9*, whose expression decreased with green space.

## 1 Introduction

Over half of the World's population, including 1.5 billion children, lives in cities with limited access to green spaces (World Cities Report, 2016; Suchitra, 2021). Green spaces have been linked to lower mortality and beneficial physical and mental health effects over the entire lifecourse (Rojas-Rueda et al., 2019; Bauwelinck et al., 2021; Astell-Burt and Feng, 2019; Twohig-Bennett and Jones, 2018; Islam et al., 2020; Barboza et al., 2021; Engemann et al., 2019; Bijnens et al., 2020). These effects are suggested to be the result of various mechanisms, including the provision of aesthetic spaces for restoring psychological stress via relaxation, increasing social contacts and physical activity, enriching microbial input from the environment (Dockx et al., 2021), reducing noise and air pollution levels, and moderating ambient temperature (Chen et al., 2021; Markevych et al., 2017). Nevertheless, these pathways do not fully explain the beneficial effects of green spaces, and in-depth knowledge of the underlying biological mechanisms is key for a better understanding.

A sensitive measure that can represent a sensor of external stimuli, interfacing between exposure and health outcome, is epigenetics (Goldberg et al., 2007). Epigenetics refers to mechanisms that can perpetuate alternative gene activity states in the context of the same DNA sequence (Chen et al., 2021). DNA methylation is the epigenetic mechanism most studied and consists of the transfer of a methyl group in the C5 position of the cytosine to form 5-methylcytosine, which can be induced by environmental factors and influence the ability of DNA to regulate gene expression (Martin and Fry, 2018). To date, green space has been associated in adults with DNA methylation (Jeong et al., 2022; Xu et al., 2021a) and with a validated DNA methylation-based epigenetic mortality risk score (Ward-Caviness et al., 2020), with DNA methylation in children of two years of age (Lee et al., 2021) and with placental DNA methylation of serotonin receptor (Dockx et al., 2022). However, DNA methylation plays a fundamental role in development already in early life, beginning *in utero* (Felix and Cecil, 2019). Whether exposure to green space during *in utero* life, which represents a susceptibility window during which epigenetic programming is sensitive to environmental influences, impacts newborns DNA methylation has not been studied yet.

Therefore in this study, we conducted the first epigenome-wide association study (EWAS) of maternal residential exposure to green space during gestation with cord blood DNA methylation regions and sites, and its downstream links with cord gene expression, by meta-analysing data from two subpopulations of the ENVIRonmental influence ON early AGEing (ENVIR*ON*AGE) birth cohort.

## 2 Material and methods

### 2.1 Study population

The study population included two subsamples of the ENVIR*ON*AGE cohort based on the availability of genome-wide DNA methylation profile in cord blood: 200 children with methylome data measured with Illumina HumanMethylation 450K BeadChip arrays selected as participating in the EXPOsOMICS European FP7 project (Vineis et al., 2017), and 377 children with methylome data measured with Illumina HumanMethylation EPIC BeadChip arrays selected as participating at the study follow-up at four years of age. ENVIR*ON*AGE is an ongoing mother-child cohort set up in Belgium, including more than 2000 singletons, and whose inclusion and recruitment details can be found elsewhere (Janssen et al., 2017). Ethical approval was obtained by the ethical committee of Hasselt University and East-Limburg Hospital (Genk, Belgium) (EudraCT B37120107805) and informed consent to use their data was obtained from all mothers. The final sample size included in the meta-analyses (N = 538) was determined by the number of participants that had complete data on confounders, outcomes and exposures in each subset (N = 189 out of 200 from the original subset with methylation measured with the HumanMethylation 450K BeadChip arrays and N = 349 out of 377 from the subset with methylation measured the EPIC array). Samples rates of missing data in each subset are reported in Supplementary Table 1.

### 2.2 Maternal exposure to green space during pregnancy

Questionnaires were used to retrieve information on the maternal residential addresses during the pregnancy, which were geocoded to calculate green space exposure at 100 m and 1000 m around the residential address. Green space was calculated on the land cover data of the high-resolution (1 × 1 m) 2013 Green Map of Flanders (Groenkaart Vlaanderen) commissioned by the Agency for Nature and Forest (Agentschap voor Natuur en Bos), using the same method described elsewhere (Dockx et al., 2021). The map identifies as green space the non-agricultural vegetation and distinguishes three measures: (1) high green space referring to vegetation height >3 m; (2) low green space referring to vegetation height <3 m; and (3) total green space referring to vegetation cover (that is the sum of 1 and 2). Geographic Information System functions (ArcGIS 10 software) were used to carry out the analyses.

### 2.3 DNA methylation measurement

Immediately after delivery cord blood samples were collected, centrifuged and buffy coats were stored at −80 °C until further analysis. Samples were shipped for measuring DNA methylation using Illumina HumanMethylation 450K BeadChip arrays (N = 200) and Illumina HumanMethylation EPIC BeadChip arrays (N = 372) at two different laboratories at two different time points. For 22 samples (included in the final analysis of the 450K samples only), DNA methylation was measured with both arrays. In accordance with another study in literature (Solomon et al., 2018), overall reproducibility, assessed per sample by Pearson's correlation (r) of methylation measurements between arrays (for the common 435,975 CpGs), was high (r range = 0.99–1.00).

#### 2.3.1 Illumina HumanMethylation 450K BeadChip arrays

200 samples were shipped on dry ice to the International Agency for Research on Cancer (Lyon, France), where DNA methylation was measured with the Infinium HumanMethylation450 BeadChip arrays (450K, Illumina Inc., San Diego, USA), as described elsewhere (Wang et al., 2020). Briefly, DNA was extracted from buffy coats (QIAamp 96 DNA Blood Kit, Qiagen 51161), quantified (Quant-iT PicoGreen dsDNA Assay Kit, Molecular Probes P7589), bisulfite converted (600 ng of DNA using EZ-96 DNA Methylation kit, Zymo Research D5004), hybridized to Illumina HumanMethylation 450 K BeadChip arrays and arrays were scanned using an Illumina iScan. Methylation features were filtered from cross-reactive probes and low-quality probes (probes having bead counts <3 in at least 5% of samples). Data quality was further assessed using box plots for the distribution of methylated and unmethylated signals, and multidimensional scaling plots and unsupervised clustering were used to check for sample outliers and potential gender mismatches, which were removed from the analysis. Also, samples having >1% of CpG sites with a detection P-value > 0.05 were removed. This resulted in a total of 197 samples with DNA methylation at 470,963 CpGs available for this study.

#### 2.3.2 Illumina HumanMethylation EPIC BeadChip arrays

377 samples were shipped on dry ice to the GenomeScan laboratory (Leiden, The Netherlands), where DNA methylation was measured by llumina HumanMethylationEPIC Bead-Chip arrays (EPIC, Illumina Inc., San Diego, USA) as described elsewhere (Wang et al., 2021). Briefly, DNA was extracted from buffy coats (QIAamp DNA mini kit, Qiagen Inc., Venlo, The Netherlands), quantified (Quant-IT assay, Thermo Fisher Scientific, USA), bisulphite converted (EZ DNA Methylation Gold kit, Zymo Research, Irvine, CA, USA), hybridized to the llumina HumanMethylationEPIC Bead-Chip arrays and scanned using an Illumina iScan. Data quality was assessed by the R script MethylAid. Based on detection p-value > 10e-16 according to Lehne et al., 2015 (Lehne et al., 2015), probe call rate was set to < 95%. No sample was deleted for low sample call rate (<98%) as no sample had more than 2% missing probes. Samples with discordant sex, predicted using shinyMethyl (Fortin et al., 2014a), were removed. This resulted in a total of 372 samples with DNA methylation at 857,898 CpGs available for further analysis. Twenty-two of these samples were excluded from analysis as they were already analysed using 450K arrays, leaving a total of 350 samples.

#### 2.3.3 Preprocessing

For each probe the methylation level was expressed as β value. For both arrays, to increase comparability, we performed functional normalization (Fortin et al., 2014b) using the minfi package in R (Aryee et al., 2014). Data were trimmed removing the outliers using Tukey's method and probes were then filtered to exclude those with missing values in more than 20% of the samples. Additionally, we excluded probes: i) located on X and Y chromosomes, ii) non-CpG targeting and iii) known to overlap known SNPs (SNPs at CpGs/single-base extension (SBE) with the minor allele frequency >0.05 were excluded), and iv) cross-reactive with multiple genomic locations via Maxprobe package in R (Pidsley et al., 2016). This resulted in a total of 423,711 CpGs for the 450K arrays and 785,952 CpGs for the EPIC arrays and 396,641 common CpGs for both the arrays.

#### 2.3.4 Cord blood cell estimation

Cord blood cell proportions (nucleated red blood cells, granulocytes, monocytes, natural killer cells, B cells, CD4^+^ T cells, and CD8^+^ T cells) were estimated using the Gervin algorithm (Gervin et al., 2019).

### 2.4 Covariates

Information on newborns' ethnicity (European if at least two grandparents were Europeans or not otherwise), maternal smoking habits (current or not) during the pregnancy, maternal educational level at birth based on educational achievement (low if mothers did not obtain any diploma, medium if the highest diploma obtained was a high school diploma, and high if the highest diploma obtained was from college or university degree), was obtained by questionnaires filled out by the mothers after the delivery.

Information on newborns' sex (boys or girls), gestational age (in weeks) based on the difference between the child's date of birth and the date of conception, which was estimated on the last menstrual period in combination with the first ultrasonographic examination, maternal age at delivery (in years) based on the difference between the mother's date of birth and the child's date of birth, parity (primiparous, secondiparous or multiparous), and season of delivery based on the date of delivery (winter if between December 21st and March 20th, spring if between March 21st and June 20th, summer if between June 21st and September 20th and autumn if between September 21st and December 20th) was collected from medical hospital records.

As detailed in the respective references, average maternal exposure to particulate matter with an aerodynamic diameter <2.5 μg/m³ (PM_2.5_) during pregnancy (in mg/m^3^) (Wang et al., 2020) and urbanicity (urban, suburban, and rural) (Vos et al., 2022) were estimated based on the maternal residential address at birth obtained from questionnaires and then geocoded. In brief, PM_2.5_ exposure was estimated using a spatiotemporal interpolation method, taking into account both land-cover data and pollution data from fixed monitoring stations in combination with a dispersion model (Janssen et al., 2008; Lefebvre et al., 2011, 2013) and daily values were averaged over the entire pregnancy (Wang et al., 2020). Urbanicity at the maternal residential address was based on the statistical sector of the Flemish Government–Department Environment map and classified as urban, suburban, and rural areas depending on population density, employment, location, and spatial planning (Vos et al., 2022).

Similarly, based on geocoded maternal residential addresses during pregnancy, we calculated the distance from major roads, namely freeways and national roads (in meters), and estimated the median maternal neighborhood income from the tax declarations per statistical sectors as disclosed by the Belgian statistical office (Statbel) which include taxable professional income, replacement income, pensions, dividends, cadastral income and maintenance payments, and by excluding non-taxable income, such as child benefits and integration income. The data reference period used was the 2014 income year, i.e., the 2015 tax year. The tax statistics were compiled according to the taxpayer's place of residence.

### 2.5 Gene expression

To better characterize the functional implication of the identified DNA methylation signatures of green space, gene expression levels were measured in cord blood samples of the ENVIR*ON*AGE cohort (N = 200) in the same subpopulation with 450K DNA methylation data available. Samples were shipped to Maastricht University, where gene expression was measured using Agilent Whole Human Genome 8 × 60 K microarrays as detailed previously (Alfano et al., 2020). In brief, RNA was extracted from buffy coats (total RNA miRNeasy mini kit, Qiagen, Venlo, Netherlands), cyanine-3 labeled (Quick-Amp Labeling Kit One-Color, Agilent Technologies), hybridized onto Agilent Whole Human Genome 8 × 60 K microarrays and scanned by Agilent DNA G2505C Microarray Scanner. An in-house developed quality control pipeline in R software was used to preprocess raw data as follows: local background correction, omission of controls, flagging of bad spots and spots with too low intensity, log_2_ transformation and quantile normalization using arrayQC. More information about the flagging and the R-scripts of the pipeline is available at https://github.com/BiGCAT-UM/arrayQC_Module. Further preprocessing included removal of probes showing >30% flagged data, merging of replicate probes based on the median, and imputation of missing values by means of K-nearest neighbour imputation (K = 15), resulting in 29,164 transcripts for 165 samples that remained available for subsequent analyses. For the functional analyses, we selected the transcripts of the genes identified to be differentially methylated with maternal exposure during pregnancy to green space in the main analysis.

### 2.6 Statistical analysis

#### 2.6.1 Epigenome-wide association studies

We first investigated the methylation at single CpG sites in relation to maternal exposure to green space during pregnancy by performing separate EWASs for samples with EPIC and 450K arrays methylation data. In each single subset, DNA methylation levels were modeled as β values representing the dependent variables in linear mixed models with fixed effects of newborns' sex, ethnicity, gestational age, season at delivery, day of sampling, parity, maternal age at delivery, maternal smoking during pregnancy, maternal educational level and estimated blood cell counts, and random effects of bead array row and bisulphite conversion batch. Quality control of the results was performed by visual inspection of scatter plots of coefficients, precision plots, quantile-quantile plots (QQ-plots) of p-values, and calculating inflation using the bacon method (λ) via the R package bacon (van Iterson et al., 2017).

#### 2.6.2 Meta-analysis of EWAS

Then, single EWAS results were meta-analysed via fixed-effects inverse-variance weighted (IVW) meta-analyses using the R package metafor. Meta-analyses were restricted to CpGs available in both cohorts (N = 396,641). Results were presented as percentage mean (and standard error) methylation value differences per one interquartile range increase in green space levels. To account for multiple testing, false discovery rate (FDR) using the Benjamini–Hochberg (BH) method was applied, with values below 0.05 considered genome-wide significant (Benjamini et al., 2001). Significance was considered suggestive for p-values lower than 1-e05. When CpG annotation to corresponding genes provided by Illumina was missing, we searched for the closest genes within 10 Mb in the UCSC Genome Browser via the FDb.InfiniumMethylation.hg19 R package. Quality control of the results was performed by visual inspection of quantile-quantile plots (QQ-plots) of p-values and calculating λ. We assessed the robustness of the findings to inter-study heterogeneity by QQ-plots of the heterogeneity statistic (I_2_) and removed CpGs with I_2_ >50% from further analyses.

#### 2.6.3 Differentially methylated regions

Differentially methylated regions (DMRs) in relation to residential green space exposure were identified using ENmix-combp and DMRcate R packages (Xu et al., 2021b; Peters et al., 2015). We used as inputs the results from the meta-EWASs (estimated coefficients, p-values and z-values) of green space and set for DMRcate the minimum number of CpGs in a region to two and the minimum length of nucleotides to 1000, and for comb-p the bin size to 310 and the seed to 0.05. To correct for multiple comparisons, we used FDR correction in DMRcate, and 1-step Siddak correction in ENmix-combp, with values below 0.05 for both algorithms considered significant. Only DMRs including at least three CpGs in a region were considered. DMRs were annotated using matchGenes function from minfi R package.

#### 2.6.4 Analysis of hypothalamic-pituitary-adrenal axis candidate genes

In order to assess if exposure to residential green space affects methylation sites previously associated with maternal stress, we performed a look-up analysis of CpGs annotated to seven hypothalamic-pituitary-adrenal (HPA) axis candidate genes (*NR3C1*, *FKBP5*, *11β-HSD2*, *CRH*, *CRHBP*, *SLC6A4*, and *OXT*) (Benjamini et al., 2001).

#### 2.6.5 Association with gene expression

To improve the interpretation of the findings, we investigated the effect of maternal green space during pregnancy on gene expression of the genes differentially methylated in the meta-EWASs. Expression levels of genes were modeled as dependent variables and green space measurements as independent variables in linear mixed models with fixed effects of newborns' sex, ethnicity, gestational age, season at delivery, day of sampling, parity, maternal age at delivery, maternal smoking during pregnancy, maternal educational level, white blood cells counts, and random effect of hybridization date. Associations were deemed significant if FDR-corrected p-values were <0.05.

#### 2.6.6 Look-up analysis of findings in literature

Finally, we compared meta-EWASs results with associations previously reported in placenta (Dockx et al., 2022), in children (Lee et al., 2021) and in adult studies (Jeong et al., 2022; Xu et al., 2021a).

#### 2.6.7 Sensitivity and robustness analyses

Sensitivity analyses were performed by repeating the meta-analyses adding as covariates to the models the following maternal exposures during pregnancy: i) PM_2.5_, ii) distance to nearest major roads, iii) urbanicity, and iv) median neighborhood income.

To test the stability of our results, we repeated all the analyses using non-trimmed DNA methylation. We also checked if results in single EWASs were consistent with meta-analysis results.

## 3 Results

**Fig. 1:**
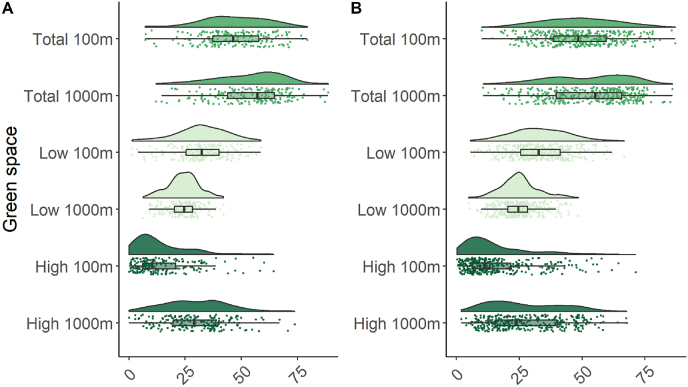
Raincloud plots combining boxplots, raw jittered data, and a split-half violin of maternal green space exposure during pregnancy for children (A) with 450K (N = 189) and (B) EPIC (N = 349) arrays cord blood methylation data available. . (For interpretation of the references to colour in this figure legend, the reader is referred to the Web version of this article.)

The meta-analysis sample included 538 newborns from two populations, 189 newborns with cord blood methylation measured with the HumanMethylation 450 KBeadChip arrays and 349 newborns with cord blood methylation measured using the EPIC arrays (Table 1). Mean maternal exposure to green space during pregnancy ranged between 15.3% for high green space at 100 m buffer to 52.9% for total green space at 1000 m buffer (Table 1). Cohort-specific distributions of maternal exposure to green space during pregnancy are shown in Fig. 1 and were not different between the two subsets (all the p-values from the Mann-Whitney-Wilcoxon test were >0.05). As expected, green space measurements in each subset were positively correlated over the different distance buffers, total green space measurements were positively correlated with low and high green space measurements over the different distance buffers, and high and low green space at 100 m and at 1000 m buffers were negatively correlated (Supplementary Fig. 1). 50.0% of the newborns were girls, their mean gestational age at birth was 39.1 weeks, and only 7.4% were non-European (Table 1). Mean maternal age was 29.9 years, 53.7% of the mothers were at their first pregnancy, delivery occurred during spring in 30.9% of the cases, 12.1% of the mothers were smokers during pregnancy, 60.2% of them had a college or university degree, mean maternal exposure to PM_2.5_ during pregnancy was 13.9 mg/m^3^, mean distance to major roads from the maternal residence was 557.9 m, 44.3% of the mother were living in a rural area and mean maternal median neighborhood income was 25246.7 euros (Table 1).

**Table 1:** Characteristics of the ENVIRONAGE study population included in the meta-analyses and of the two subsets with 450K and EPIC DNA methylation data.

	450K arrays samples N = 189	EPIC arrays samples N = 349	Meta-analysis N = 538
Newborns' sex, girls	90 (47.62)	179 (51.29)	269 (50.00)
Gestational age, weeks	39.10 (1.67)	39.14 (1.61)	39.12 (1.63)
Ethnicity, non-European	19 (10.05)	21 (6.02)	40 (7.43)
Maternal age, years	29.36 (4.32)	30.15 (4.30)	29.87 (4.32)
Parity,			
*Primiparous*	103 (54.50)	186 (53.30)	289 (53.72)
*Secundiparous*	58 (30.69)	125 (35.82)	183 (34.01)
*Multiparous*	28 (14.81)	38 (10.89)	66 (12.27)
Season at delivery,			
*Winter*	43 (22.75)	95 (27.22)	138 (25.65)
*Spring*	80 (42.33)	86 (24.64)	166 (30.86)
*Summer*	25 (13.23)	85 (24.36)	110 (20.45)
*Autumn*	41 (21.69)	83 (23.78)	124 (23.05)
Maternal smoking, yes	25 (13.23)	40 (11.46)	65 (12.08)
Maternal education,			
*Low*	26 (13.76)	25 (7.16)	51 (9.48)
*Medium*	65 (34.39)	98 (28.08)	163 (30.30)
*High*	98 (51.85)	226 (64.76)	324 (60.22)
PM_2.5,_ mg/m^3^	12.94 (1.76)	14.46 (2.48)	13.92 (2.37)
Distance to nearest major roads, meters	587.39 (653.31)	544.42 (609.33)	557.87 (624.28)
Urbanicity			
*Urban*	77 (40.74)	112 (32.09)	189 (35.20)
*Suburban*	27 (14.29)	83 (23.78)	110 (20.48)
*Rural*	85 (44.97)	154 (44.13)"	238 (44.32)
Median neighborhood income, euros	24775.68 (3483.94)	25500.39 (3121.03)	25246.67 (3267.82)
Maternal exposure to green space during pregnancy			
*Total 100m*	46.78 (14.62)	48.76 (14.74)	48.07 (14.72)
*Total 1000m*	53.87 (15.09)	52.48 (15.88)	52.97 (15.61)
*Low 100m*	32.25 (11.21)	32.98 (11.00)	32.72 (11.07)
*Low 1000m*	24.16 (6.84)	25.00 (7.30)	24.71 (7.15)
*High 100m*	14.53 (12.66)	15.78 (13.32)	15.34 (13.09)
*High 1000m*	29.71 (14.00)	27.47 (14.68)	28.26 (14.47)

Number (%) and mean (±standard deviation) are reported for categorical and continuous variables, respectively. *p-values from chi-square and *t*-test statistics are reported for categorical and continuous variables.

### 3.1 Meta epigenome-wide association studies

**Fig. 2:**
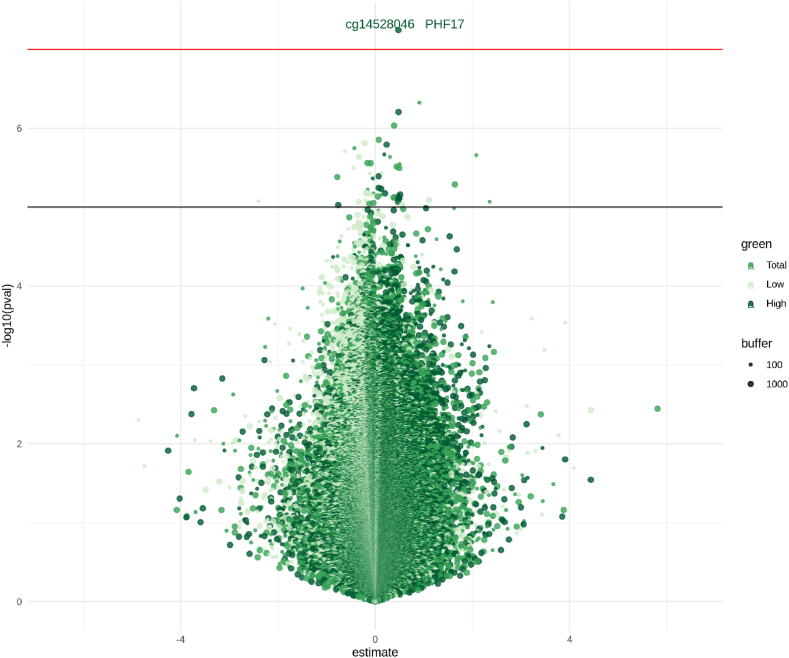
Volcano plot shows estimates on x-axis and (-log10) p-values on y-axis for associations of the meta-EWASs of total, low and high maternal green space at 100 m and 1000 m buffers distance. The horizontal red line represents FDR corrected p-value threshold of 0.05, the horizontal black line represents p-value threshold of 1-e05. (For interpretation of the references to colour in this figure legend, the reader is referred to the Web version of this article.) Estimates represent % increase of DNA methylation per one interquartile range increase of maternal exposure to green space during pregnancy.

One CpG (cg14528046, located on *PHF17*) was genome-wide significant in the meta-EWASs (Fig. 2 and Supplementary Fig. 2), while 72 CpGs were suggestive significant (p-values<1e-05) (Supplementary Table 2). QQ-plots of p-values did not identify any major problem (λ range = 0.98–1.06, Supplementary Fig. 3). Meta-EWASs estimates over the different green space distance buffers were positively correlated, except for estimates of high and low green measurements that were negatively correlated, consistently with what is shown in Supplementary Fig. 1 (Supplementary Fig. 4). QQ-plots of I_2_ p-values showed evidence of between-study heterogeneity (Supplementary Fig. 5) and CpGs having I_2_ > 50 were excluded from subsequent analyses (N range of excluded CpGs = 67,626–94,876, Supplementary Table 3), which resulted in a total of 54 CpGs retained in the meta-EWASs that were suggestive significant (p-values<1e-05) (Fig. 2).

**Fig. 3:**
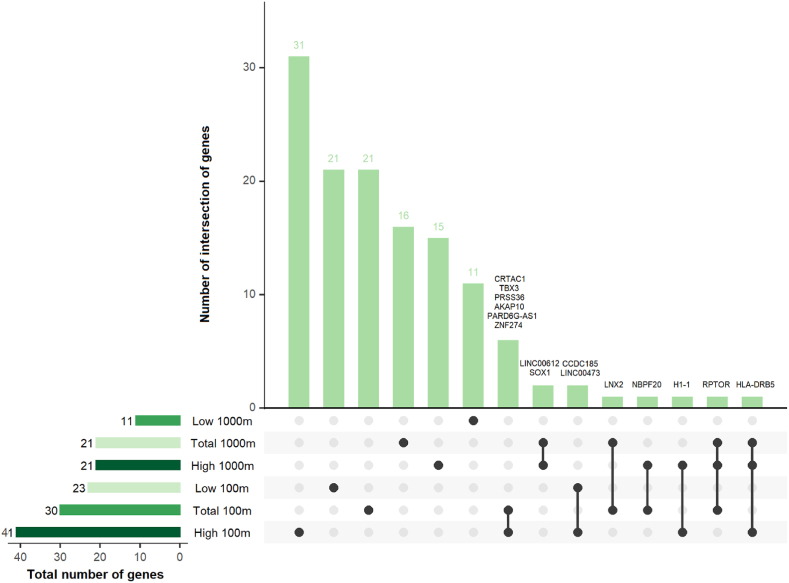
Upset plot represents the genes mapped to the differentially methylated regions in the meta-EWASs of maternal exposure to total, low and high green space during pregnancy at 100 m and 1000 m buffers and their intersections. (For interpretation of the references to colour in this figure legend, the reader is referred to the Web version of this article.)

147 cord blood differentially methylated regions (DMRs), annotated to 129 unique nearest genes, were associated with total, high and low green space residential green space exposure of the mother during pregnancy (complete list available in Supplementary Table 4)**.** As shown in Fig. 3 and 13 genes annotated to the DMRs were found commonly by two meta-EWASs, while two (*HLA-DRB5* and *RPTOR*) were found commonly by three meta-EWASs.

Quality control of EWASs conducted in each separate subset (450K and EPIC) did not identify any major problem. Observed p-values in the quantile-quantile plot in the EWASs appeared to be mostly higher than expected in the 450K samples (λ range = 0.99–1.17) (Supplementary Fig. 6) and lower than expected in the EPIC samples (λ range = 0.90–0.97) (Supplementary Fig. 7). EWASs estimates precision was higher in the EPIC subset, as testified by smaller median standard errors (Supplementary Fig. 8). In both subsets, the distribution of the estimates was not skewed (Supplementary Fig. 9).

### 3.2 Analysis of HPA axis candidate genes

We selected 145 CpGs annotated to seven HPA axis candidate genes (*NR3C1*, *FKBP5*, *11β-HSD2*, *CRH*, *CRHBP*, *SLC6A4*, and *OXT,* Supplementary Table 5). Using FDR-adjusted p-value threshold of 0.05, none of these CpG was associated with maternal exposure to green space during pregnancy (Supplementary Fig. 10).

### 3.3 Association with gene expression

We identified one cord blood transcript associated with maternal green space exposure during pregnancy that was downstream of the identified DNA methylation signals.

**Fig. 4:**
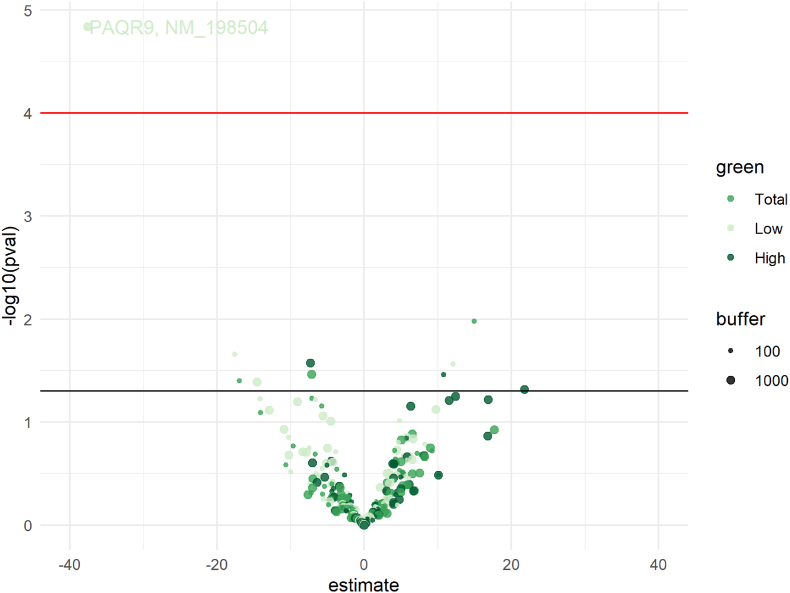
Volcano plot shows estimates on x-axis and (-log10) p-values on y-axis for associations of transcripts (corresponding to genes annotated to CpGs associated with maternal exposure to green space during pregnancy in the meta-EWASs with p-values<1e-05) and total, low and high green space during pregnancy at 100 m and 1000 m buffers (N = 157). Dots above the horizontal red line represent CpGs with FDR corrected p-values <0.05, for which corresponding gene names and mRNA accession numbers are reported. The horizontal black line represents p-value threshold of 0.05. (For interpretation of the references to colour in this figure legend, the reader is referred to the Web version of this article.) Estimates represent the log2 fold change of transcripts per one interquartile range increase of exposure to maternal exposure to green space during pregnancy.

First, the 54 CpGs associated in the meta-EWASs with green space at p-value below 1e-05 were annotated to 38 genes. Expression levels were available in the 450 K samples for 31 out of these 38 genes, for a total of 45 transcripts. Among them, *PAQR9* was differentially expressed with maternal low green space at 1000 m buffer during pregnancy at FDR p-value < 0.05 (p-value = 1.45e-05) (Fig. 4).

Second, the 147 DMRs associated with green space in meta-EWASs were annotated to 129 nearest genes. Out of these 129 genes, expression levels were available in the 450K samples for 79 genes for a total of 174 transcripts. Using FDR-adjusted p-value threshold of 0.05, no transcript was associated with maternal exposure to green space during pregnancy (Supplementary Fig. 11).

### 3.4 Look-up analysis of associations of DNA methylation and green space in literature

The look-up analysis showed nine genes annotated to significant DMRs in our analyses in cord blood overlapped previous findings in the literature (Supplementary Fig. 12).

In the same population here under study, in a targeted study, Dockx et al. (2022) found green space associated with placental methylation of *HTR2A,* which was also among the cord blood DMRs we identified as linked with green space.

Lee et al. (2021), after selection of candidate DNA methylation markers from literature, found 25 CpGs in peripheral blood from 2 years old children associated with residential surrounding greenness. These CpGs were annotated to 22 genes, none of which was among the cord blood DMRs identified by our meta-EWAS.

In adults, Jeong et al. (2022) did not identify any genome-wide significant CpGs, but 163 and 56 DMRs associated with green space at 30 and 500 m, respectively, mapped to a total of 204 genes, eight of which (*PRSS36*, *ZNF274*, *DUSP22*, *SLC38A4*, *SPON2*, *COL11A1*, *A1BG-AS1* and *KCNQ1DN*) were common to genes mapped to DMRs identified by our meta-EWAS. Further, Xu et al. (2021a) found 35 significant DMRs related to surrounding greenness, mapped to 20 nearest genes, none of which was differentially methylated in our analysis (Supplementary Fig. 12).

### 3.5 Sensitivity analyses

When adding as covariates to the models one other maternal exposure during pregnancy at a time, being i) PM_2.5_, ii) distance to nearest major roads, and iii) median neighborhood income, we found that FDR p-value of cg14528046 (located on *PHF17*), that was genome-wide significant CpG in main analysis of exposure during pregnancy to high green space at 1000 m buffer, was still below 0.05 (Supplementary Table 6). Adding urbanicity (coded as urban, suburban, and rural) as covariate FDR p-value faded above 0.05 (Supplementary Table 6). Out of the 147 identified in the meta-EWASs, 85 were still significant in all the sensitivity analyses (Supplementary Fig. 13). Among the nine genes mapped to the DMRs we identified in the main meta-EWASs in common with previous literature findings (Supplementary Fig. 12), six (*PRSS36*, *ZNF274*, *KCNQ1DN*, *COL11A1*, *A1BG-AS1* and *HTR2A*) were still significant in all the sensitivity analyses.

### 3.6 Robustness analyses

To check the robustness of our results, we repeated the analyses without trimming DNA methylation data. The association of gestational exposure to high green space at 1000 m buffer with cg14528046 was not genome-wide significant (Supplementary Table 7), while the DMRs’ results were similar to the main analyses (100 out of the 147 DMRs were still significantly associated in non-trimmed meta-EWASs, Supplementary Fig. 14). We verified if the results of the single EWASs were consistent with meta-analyses results. The association of gestational exposure to high green space at 1000 m buffer with cg14528046 was not genome-wide significant in the separate subsets (Supplementary Table 7). Among the 147 DMRs identified in the meta-EWASs, one DMR (annotated to and *ZNF27*4) was commonly significant below the FDR corrected p-value threshold of 0.05 and the direction of mean methylation of its CpGs was consistent in 450K and EPIC separate EWAS (Supplementary Table 8).

## 4 Discussion

This study shows associations between maternal exposure to green space during pregnancy and cord blood DNA methylation in two subsets of the same ENVIR*ON*AGE mother-child cohort. Maternal exposure to total, high (vegetation higher than 3 m) and low (vegetation lower than 3 m) green space during pregnancy was associated with 147 differentially methylated regions. Associations with 85 of these regions were still significant upon adjustment for maternal exposure to PM_2.5_, residential distance to major roads, urbanicity and neighborhood income during pregnancy. A suggestive association with maternal exposure to green space during pregnancy was found for 54 CpGs with p-value <1-e05, one of which, cg14528046 (located on *PHF17*), was genome-wide significantly associated with gestational exposure to high green space at 1000 m buffer (p-value = 5.68e-08). Translating these results to the gene expression level showed that the expression level of *PAQR9* (hypermethylated with maternal exposure to total green space at 100 m buffer during pregnancy) was decreased with maternal exposure to low green space at 1000 m buffer during pregnancy.

Among the genes mapped to the 147 identified DMRs, two were associated with more than two green space buffers, the major histocompatibility complex, class II, DR beta 5 (*HLA-DRB5*) and the regulatory associated protein of mTOR complex 1 (*RPTOR*) (Fig. 3) and remained significant in sensitivity analyses. *HLA-DRB5* encodes a protein involved in the immune response by presenting peptides derived from extracellular proteins, and its DNA methylation pattern has also been associated with neurological diseases, including autism spectrum disorder and Alzheimer (Sun et al., 2022; Yu et al., 2015). RPTOR negatively regulates the mTOR complex 1 pathway, which plays roles in cell growth and immune responses and impacts dopamine neuron structure, as recently demonstrated in animal studies (Kosillo et al., 2022), and its DNA methylation has been associated with cardiovascular diseases and cancers (Zhu et al., 2022; Palou-Márquez et al., 2021). Further, nine identified genes have been previously found to be differentially methylated with green space in literature (Supplementary Fig. 12), including two long non coding RNAs (lncRNAs) genes: the KCNQ1 downstream neighbour (*KCNQ1DN)* and the A1BG antisense RNA 1 (*A1BG-AS1*) genes; and seven protein-coding genes: the dual specificity phosphatase 22 (*DUSP22*), the solute carrier family 38 *member* 4 (*SLC38A4*), the spondin 2 (*SPON2*), the 5-hydroxytryptamine receptor 2 A (*HTR2A),* the zinc finger protein 274 (*ZNF27*4), the collagen type XI alpha 1 chain (*COL11A1*) and the serine protease 36 (*PRSS36)* genes. Three of these observations, which also remained significant in sensitivity analyses, require further emphasis. First, DNA methylation at the regions located on the latter three protein coding genes (*ZNF27*4, *COL11A1* and *PRSS36*), which we found associated with maternal exposure during pregnancy at 100 m buffers (*ZNF27*4 and *PRSS36* were positively associated with total and high green space during pregnancy, and *COL11A1* was negatively associated with low green space), has been previously associated with normalized difference vegetation index (NDVI) at 30 m (positively with *ZNF27*4 and *PRSS36*) and at 500 m buffers (negatively with *COL11A1*) in an adult population (Jeong et al., 2022). *ZNF274* encodes a Kruppel-associated box zinc finger protein that contains 5 C2H2 zinc finger domains and is a transcriptional repressor involved in a wide range of processes (Valle-Garcia et al., 2016), including repression of the Prader-Willy syndrome maternal gene expression in neurons (Langouet et al., 2020). *COL11A1* encodes a collagen protein mainly expressed in the cartilage, which has been associated with bone disorders and cancers (Nallanthighal et al., 2021). *PRSS36* encodes a serine protease, which has been associated with Alzheimer's disease by genome-wide association studies (GWAS) (Jansen et al., 2019) and by integrating GWAS and expression quantitative trait locus data (Lee et al., 2022). Second, in the same cohort as here under study, maternal exposure to total green space during pregnancy at 1000 m, 2000 m and 3000 m buffers were associated with an increase of placental methylation of *HTR2A*, within the same region that we found negatively associated in cord blood with maternal exposure to total and high green space at 1000 m buffers during pregnancy. *HTR2A* encodes the 5-hydroxytryptamine (also known as serotonin) receptor 2 A, which plays a crucial role in fetal brain development and adult cognitive functions (Parade et al., 2017) and whose DNA methylation in saliva has been associated with posttraumatic stress disorder and major depressive disorder symptoms in preschoolers (Parade et al., 2017). Taken together, these findings suggest a possible underlying mechanism of identified signals in the effects of maternal exposure to green space during pregnancy on neurodevelopment in offspring. Third, we identified DNA methylation of two lncRNAs genes being associated with maternal exposure to low (*KCNQ1DN*, showing a negative association) and high (*A1BG-AS1,* showing a positive association) green space at 1000 m buffer during pregnancy and remaining significant in all sensitivity analyses, which has also been previously positively associated with NDVI at 30 m in adults (Jeong et al., 2022). *KCNQ1DN* is an imprinting gene that is expressed in the maternal allele. *KCNQ1DN'*s DNA methylation is associated with aging (Koch and Wagner, 2011) and its lncRNA was found downregulated in renal cell carcinoma in a *in vitro* study (Yang et al., 2019). Similarly, *A1BG-AS1* is overexpressed in different cancers, including hepatocellular and breast carcinoma (Bai et al., 2019; Cai et al., 2021). Other than a specific underlying mechanism of identified signals in the effects of prenatal exposure to green space on cancerogenesis, we speculate that these results may indicate a possible involvement in cell proliferation occurring during the normal development of the offspring.

Similar to previous studies in adults, the DMR-analyses derived more significant signals as compared to the single CpG-analyses (Jeong et al., 2022; Xu et al., 2021a). This may be due to biological reasons such as the high correlation between neighboring CpGs or a more complex functioning of DNA methylation at the regional level rather than at single sites or to higher statistical power given the lower number of tests performed when grouping CpGs in DMRs analysis. Also, DMR analysis tends to identify regions where the CpGs do not necessarily have individual large effect sizes, but most of the time small but significant effects. Only one CpG (cg14528046) located on PHD finger protein 1 (*PHF17)* was genome-wide significant in meta-EWAS of high green space at 1000 m buffer and remained significant in all sensitivity analyses, apart from the analysis including urbanicity as covariate*. PHF17* encodes for the Jade-1 protein, which facilitates histone acetylation, is involved in cell cycle regulation and cytokinesis, may play a role in cancers, and has been associated by GWAS with the plasma triglyceride response to omega-3 fatty acid supplementation (Panchenko, 2016; Vallée Marcotte et al., 2016). The paucity of findings at single CpG sites could be due to low reproducibility at specific individual CpG sites between the two arrays (in the 22 samples with DNA methylation measured with both arrays, 36 of the 54 suggestive significant CpGs had r < 0.50), despite the high overall reproducibility per-sample (r range = 0.99–1.00). Additionally, differences between the two subsets may have influenced the results. However, green space exposure did not differ between the two subsets and results were adjusted on covariates differing in the two subsets.

Exploring the functional relevance of our results, we found that maternal exposure to low green space at 1000 m during pregnancy was associated with reduced expression of the class II progestin and adipoQ receptor 9 (*PAQR9)* gene. PAQR9*,* commonly known as membrane progesterone receptor ε (mPRε), is a non classical progesterone receptor in the nervous system (Petersen et al., 2013) which is involved in the neuroprotective effects of progesterone and its metabolite allopregnanolone (Thomas et al., 2022). When ectopically expressed out of neurons, mPRε has been found to be mainly localized in the endoplasmic reticulum and to play a role in the regulation of protein quality control, which in turn is linked to numerous human diseases, including neurodegenerative disorders (You et al., 2020). Therefore, this result points toward a possible functional relevance of our results and corroborates a possible involvement of identified signals in the effects of maternal exposure during pregnancy to green space on offspring neurodevelopment.

The mechanisms through which maternal exposure to green space during pregnancy affects DNA methylation of offspring are unknown, but possible pathways have been suggested (Lee et al., 2020; Akaraci et al., 2020; Agay-Shay et al., 2019). In our study, green space was defined as green land cover at two different distances from maternal residential addresses, within 100 m and 1000 m buffers. Maternal exposure to green space within 1000 may influence health through a variety of pathways, including access to parks during pregnancy for physical activity or increasing social contacts, and reduction of air pollution, heat and traffic noise from trees or open green spaces. On the other hand, green space exposure within 100 m may not only buffer the impact of air pollution, temperature and noise, but also act via other pathways, such as reducing maternal psychological stress by direct view of green spaces, as previously found in adults (Brace et al., 2020). Our results showed more significant DMRs with green space measurements within the 100 m buffer than the 1000 m buffer (Fig. 3), supporting the hypothesis of a visual impact of the green space seen from home. However, our results do not support that maternal stress is involved in the chain of events leading from exposure to green space to the effects on offspring DNA methylation, as we did not find any significant association in HPA axis candidate genes. The aforementioned exposures (e.g., air pollution, heat, traffic and stress) could further be confounders rather than mediators of the association of green space and offspring DNA methylation (Klompmaker et al., 2019). Our results were adjusted for maternal education, which is the dimension of socioeconomical position that most strongly predicts health in early life by reflecting maternal health behavior (Bloomberg et al., 1994). However, socioeconomic resources, such as income, could have confounded our associations between maternal exposure to green space during pregnancy and DNA methylation. Although understanding these relationships is beyond the purpose of our study, sensitivity analyses adding maternal exposure to air pollution, distance from major roads, urbanicity and median neighborhood income during pregnancy as covariates to the models demonstrated that some signals (85 DMRs) were independent of these exposures, while others were not (62 DMRs).

The strengths of this study lie in: i) the estimation of green space exposure from land cover data based on high-resolution (1 × 1 m) aerial ortho-photos; ii) the measurement of DNA methylation using two different arrays (EPIC and 450 K); iii) the testing of the results in two independent subsets (nevertheless selected from the same population); iv) the use of two different dimension reduction techniques to seek for DMRs (ENmix-combp and DMRcate R packages); v) the downstream translation of our results to the gene expression level and vi) and the differentiation between high and low green space based on the height of the vegetation, which may influence health with different mechanisms, even though we were not able to find distinct patterns.

Results should be interpreted with caution due to some limitations. Pregnancy exposure to green space was estimated based on green spaces around the maternal residential address reported at delivery without considering the time spent indoors at home or at the workplace. While our study focuses on the “quantity” of exposure to green space during pregnancy, the “quality”, intended as the variety of species of plants, has also been shown to be an important driver of the effects of green space on health (Zhang et al., 2017). We used DNA methylation in cord blood as an accessible collectible tissue for cohort birth populations, but we acknowledge that cord blood is a mix of cell types. To account for cell variability, we adjusted our analyses for estimated cell counts using a reference method specific to the umbilical cord (Gervin et al., 2019). In addition, since previous studies suggested that exposure to green space during pregnancy is beneficial for cognitive development in children (Bijnens et al., 2020), other tissues, such as nervous tissue, may better capture the effects of green space on molecular targets at birth. Meta-analysing the results from the two subsets improved the statistical power, albeit limiting the number of CpG to those included in the 450K array. The EPIC array includes the double of the 450K array's CpGs. However, it still represents only a small proportion (∼3%) of the 28.3 million of total CpG sites in the genome (Luo et al., 2014).

Since DNA methylation patterns are established, through *de novo* and demethylation events, during pregnancy and persist in later life, the epigenetic fingerprint of green space at birth identified in this study deserves further investigation as it might have long-term consequences on health and diseases.

To our knowledge, this is the first study exploring the relationship between maternal exposure to green space during pregnancy and epigenome-wide DNA methylation at birth. Our results demonstrate that exposure to residential green space during pregnancy is associated with alterations in cord blood DNA methylation, mainly on those loci that are organized in regions. Exploring the functional relevance of our results, we found differential PAQR9 gene expression associated with maternal exposure to green space. The prenatal period represents an important window of vulnerability for brain development as well as epigentic programming, various of the identified genes, including the serotonin receptor 2 A, play important roles in neurological development and may represent an underlying mechanism of the influence of green space on neurological pathways.

## Credit author statement

RA: Conceptualization, Methodology, Formal analysis, Writing - Original Draft; EB: Conceptualization, Writing - Review & Editing; SASL: Writing - Review & Editing; TN: Conceptualization, Writing - Review & Editing, Resources, Supervision; BR: Writing - Review & Editing; KV: Writing - Review & Editing; CW: Writing - Review & Editing, MP: Conceptualization, Writing - Original Draft, Resources, Supervision.

## Funding

This work is supported by the Bijzonder Onderzoeksfonds 10.13039/501100009550Hasselt University through PhD fellowships [to RA, BR, CW], the Flemish Scientific Fund (G059219N) [to KV] and the Marguerite-Marie Delacroix foundation [to EMB] and the province of Limburg. The ENVIR*ON*AGE cohort is supported by the 10.13039/100010663EU Research Council “project ENVIR*ON*AGE” (ERC-2012-StG 310,890), the Flemish Scientific Fund (G073315N/G048420N/G026222N) and the Methusalem funding. The generation of DNA methylation data have been additionally supported from the EXPOsOMICS project and the Research Foundation – 10.13039/501100011878Flanders (10.13039/501100003130FWO) grant 1523817N [to SL].

## Declaration of competing interest

The authors declare that they have no known competing financial interests or personal relationships that could have appeared to influence the work reported in this paper.

## Data Availability

450K DNA methylome and trascriptome data are available via GEO repository with the Accession No GSE151042 and GSE151373, respectively. EPIC DNA methylome data is available upon request.
