# Supplementary material for: Epigenome-wide analysis of maternal exposure to green space during gestation and cord blood DNA methylation in the ENVIRONAGE cohort
Source: Environ Res. 2023 Jan 1;216:114828. doi: 10.1016/j.envres.2022.114828 (PMC9760568; doi:10.1016/j.envres.2022.114828)
Supplement: Multimedia component 1 [file mmc1.docx]

Supplementary information to

Epigenome-wide analysis of maternal exposure to green space during gestation and cord blood DNA methylation in the ENVIR*ON*AGE cohort

Alfano R et al.

Contents

[Supplementary Figure 1 2](#_Toc118991777)

[Supplementary Figure 2 3](#_Toc118991778)

[Supplementary Figure 3 4](#_Toc118991779)

[Supplementary Figure 4 5](#_Toc118991780)

[Supplementary Figure 5 6](#_Toc118991781)

[Supplementary Figure 6 7](#_Toc118991782)

[Supplementary Figure 7 8](#_Toc118991783)

[Supplementary Figure 8 9](#_Toc118991784)

[Supplementary Figure 9 10](#_Toc118991785)

[Supplementary Figure 10 11](#_Toc118991786)

[Supplementary Figure 11 12](#_Toc118991787)

[Supplementary Figure 12 13](#_Toc118991788)

[Supplementary Figure 13 14](#_Toc118991789)

[Supplementary Figure 14 16](#_Toc118991790)

[Supplementary Table 1 17](#_Toc118991791)

[Supplementary Table 2 18](#_Toc118991792)

[Supplementary Table 3 20](#_Toc118991793)

[Supplementary Table 4 21](#_Toc118991794)

[Supplementary Table 5 25](#_Toc118991795)

[Supplementary Table 6 26](#_Toc118991796)

[Supplementary Table 7 27](#_Toc118991797)

[Supplementary Table 8 28](#_Toc118991798)

## Supplementary Figure 1

Heatmaps represent Spearman's correlation matrices of maternal exposure to green space during pregnancy for children (**A**) with 450K and (**B**) EPIC arrays methylation data available. P-values for all the correlations are <0.05.


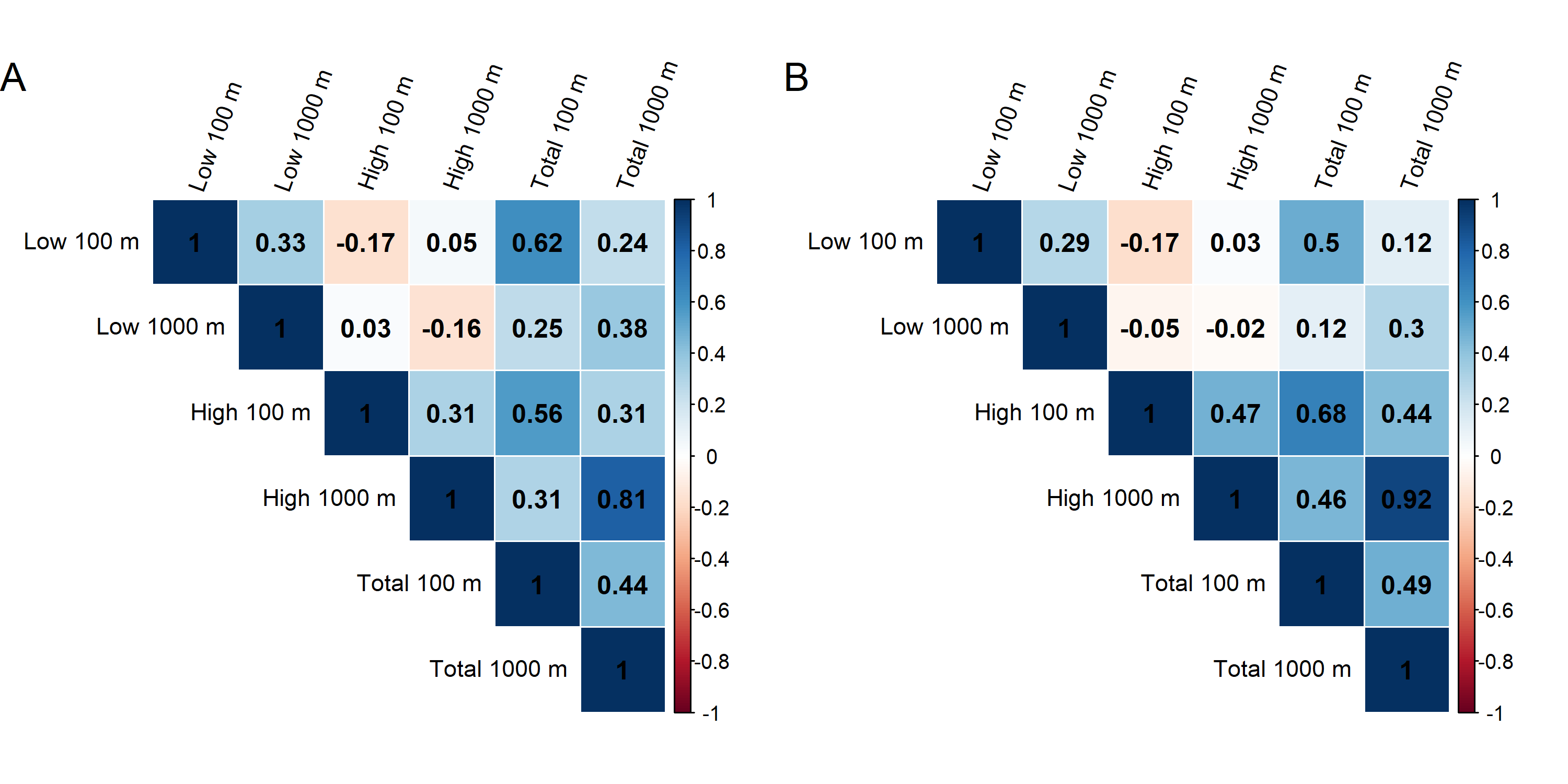


## Supplementary Figure 2

Manhattan plots of p-values of meta-EWASs of maternal green space during pregnancy.


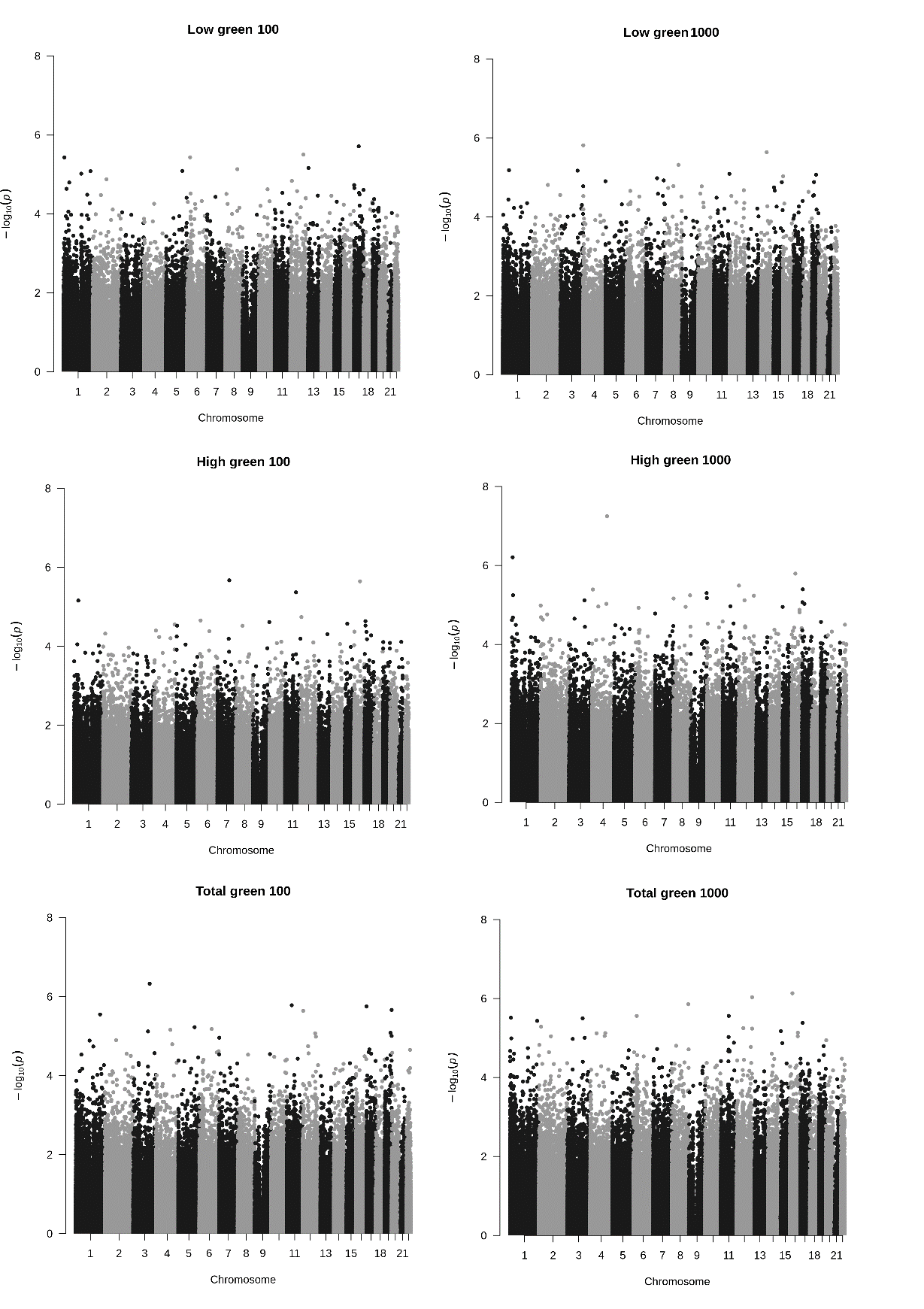


## Supplementary Figure 3

Quantile-quantile (QQ) plots of p-values of meta-EWASs of maternal green space during pregnancy.


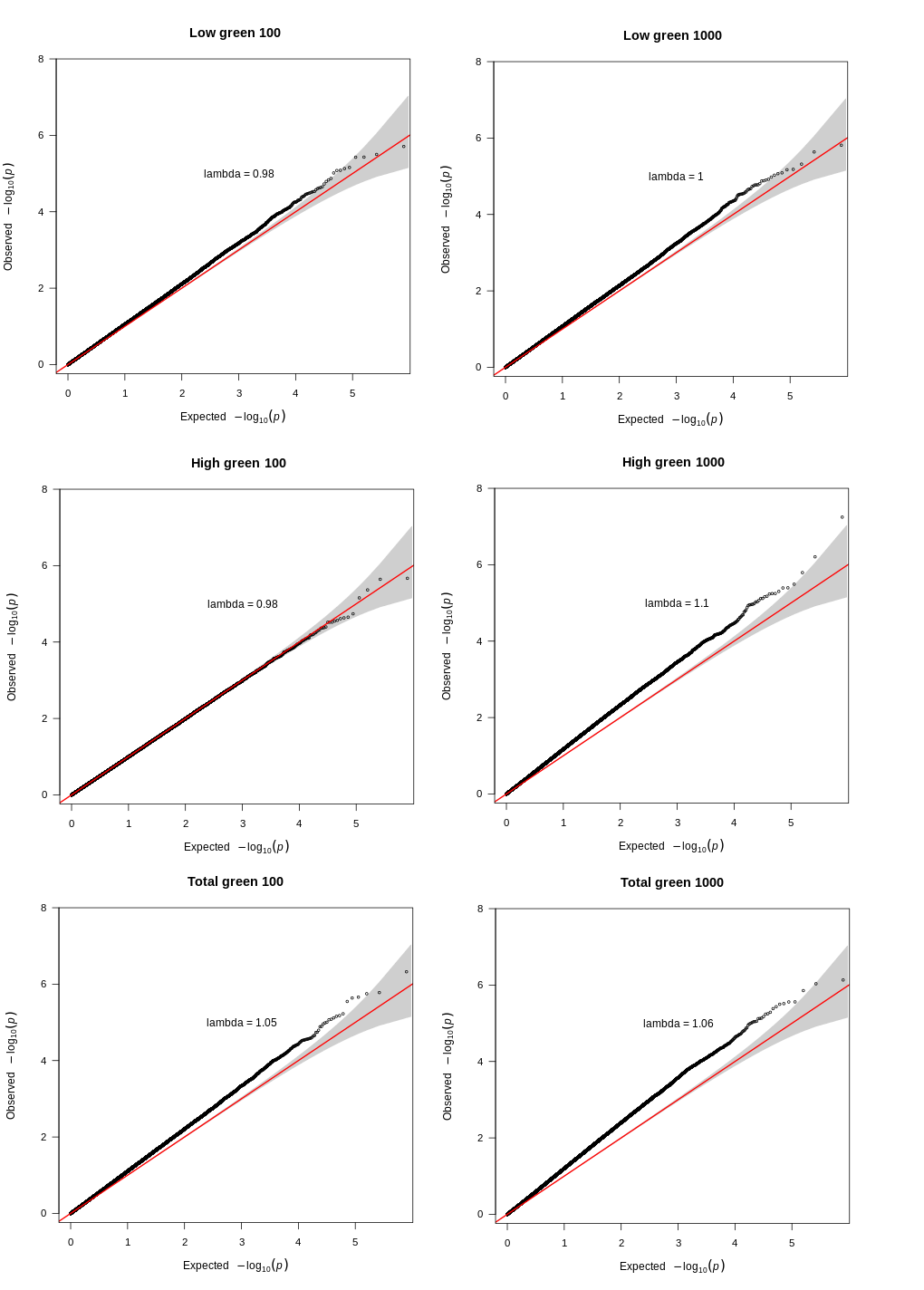


## Supplementary Figure 4

Heatmaps represent Pearson's correlation matrices of estimates from meta-EWASs of maternal green space during pregnancy.


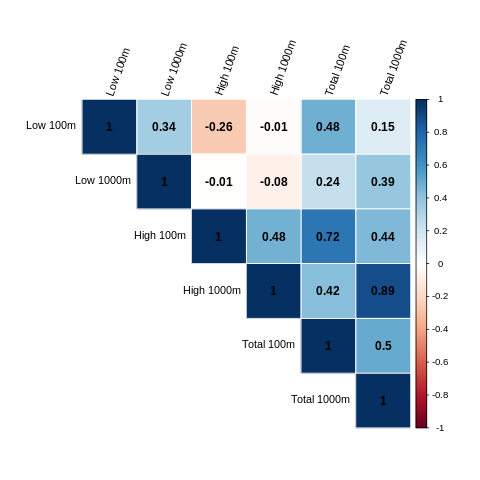


## Supplementary Figure 5

Quantile-quantile (QQ) plots of I_2_ p-values of meta-EWASs of maternal green space during pregnancy.


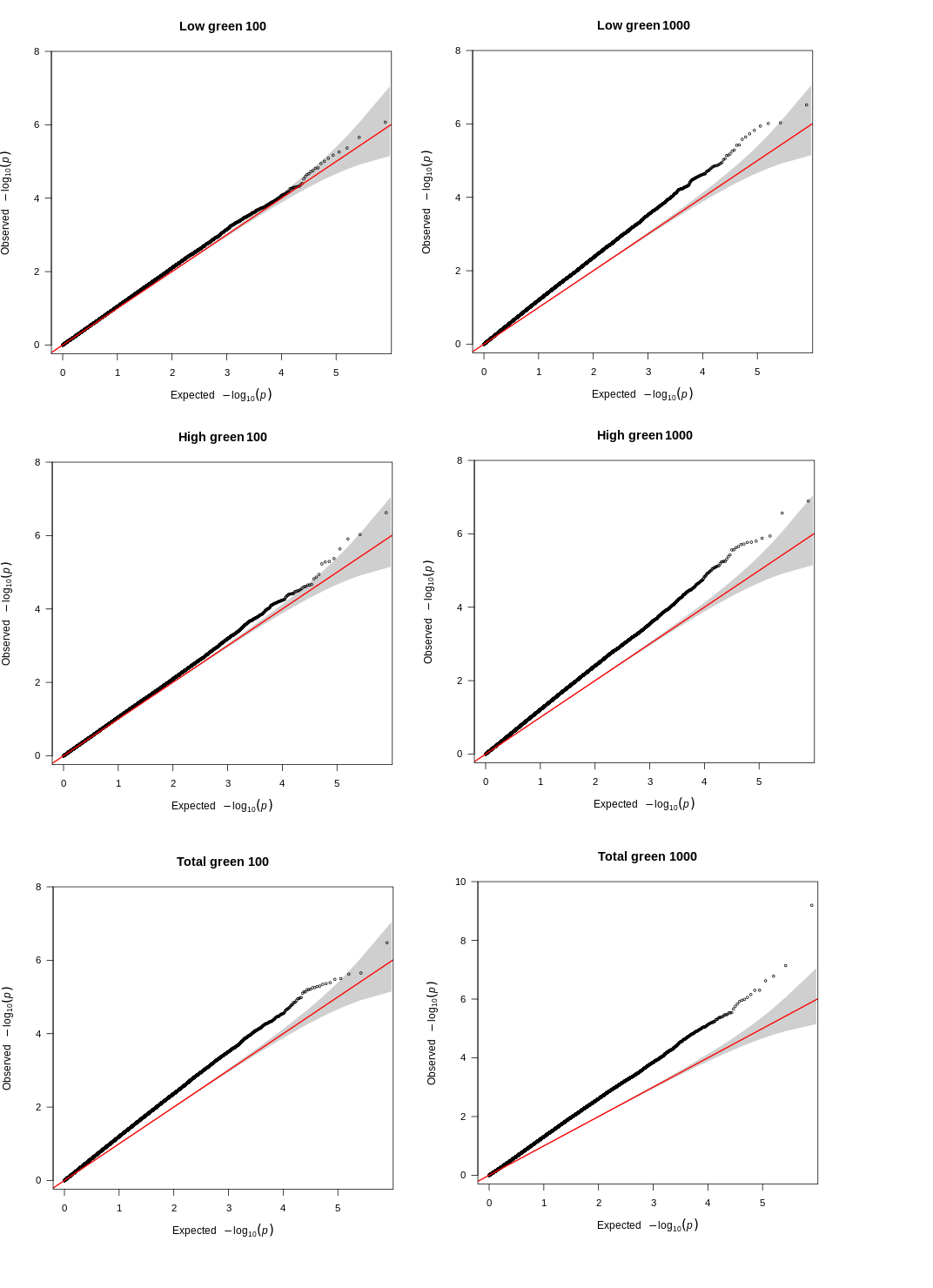


I_2_= heterogeneity

## Supplementary Figure 6

Quantile-quantile (QQ) plots of p-values from single EWASs of maternal green space during pregnancy for children with 450K methylation data available.


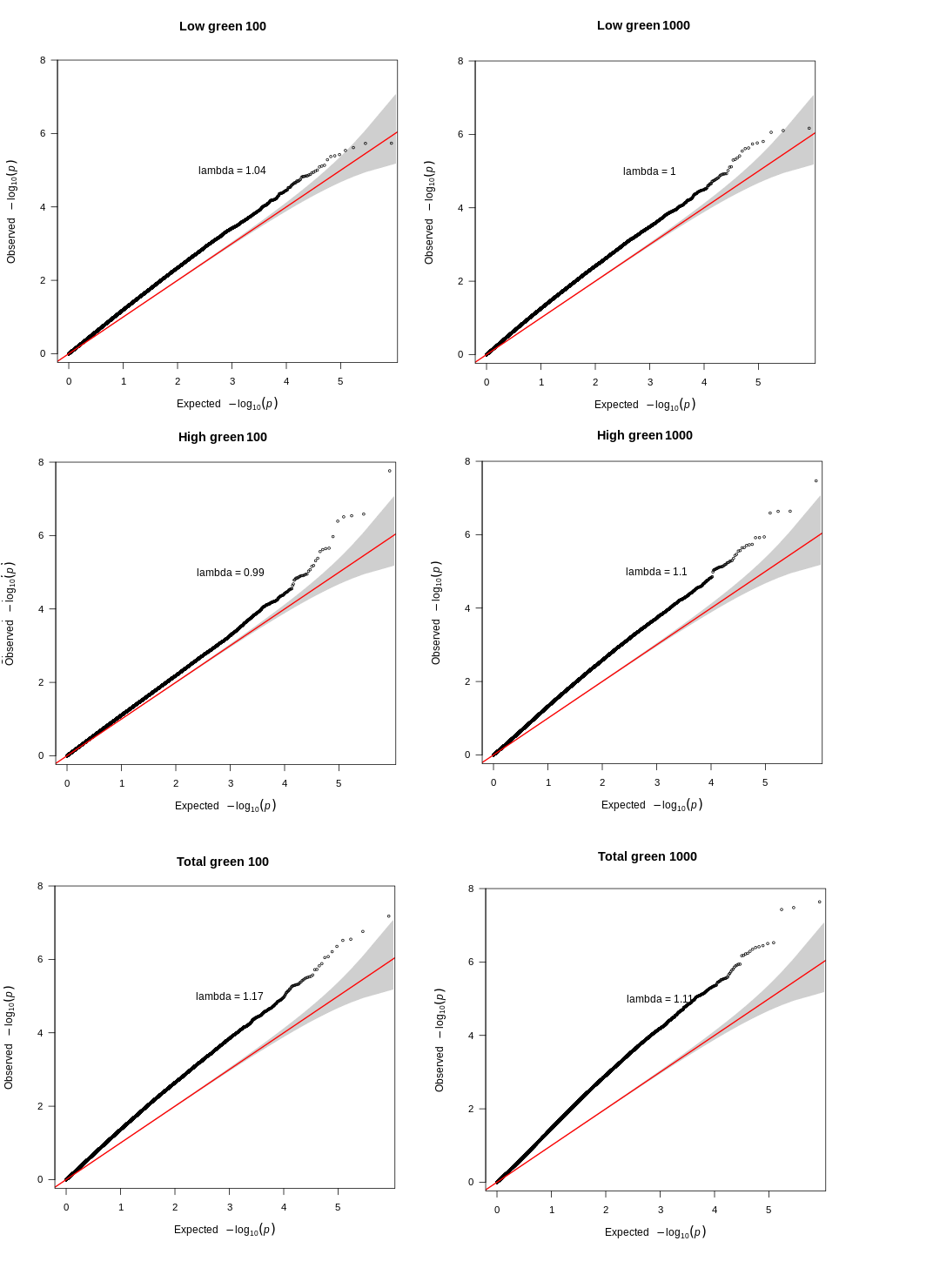


## Supplementary Figure 7

Quantile-quantile (QQ) plots of p-values from single EWASs of maternal green space during pregnancy for children with EPIC methylation data available.


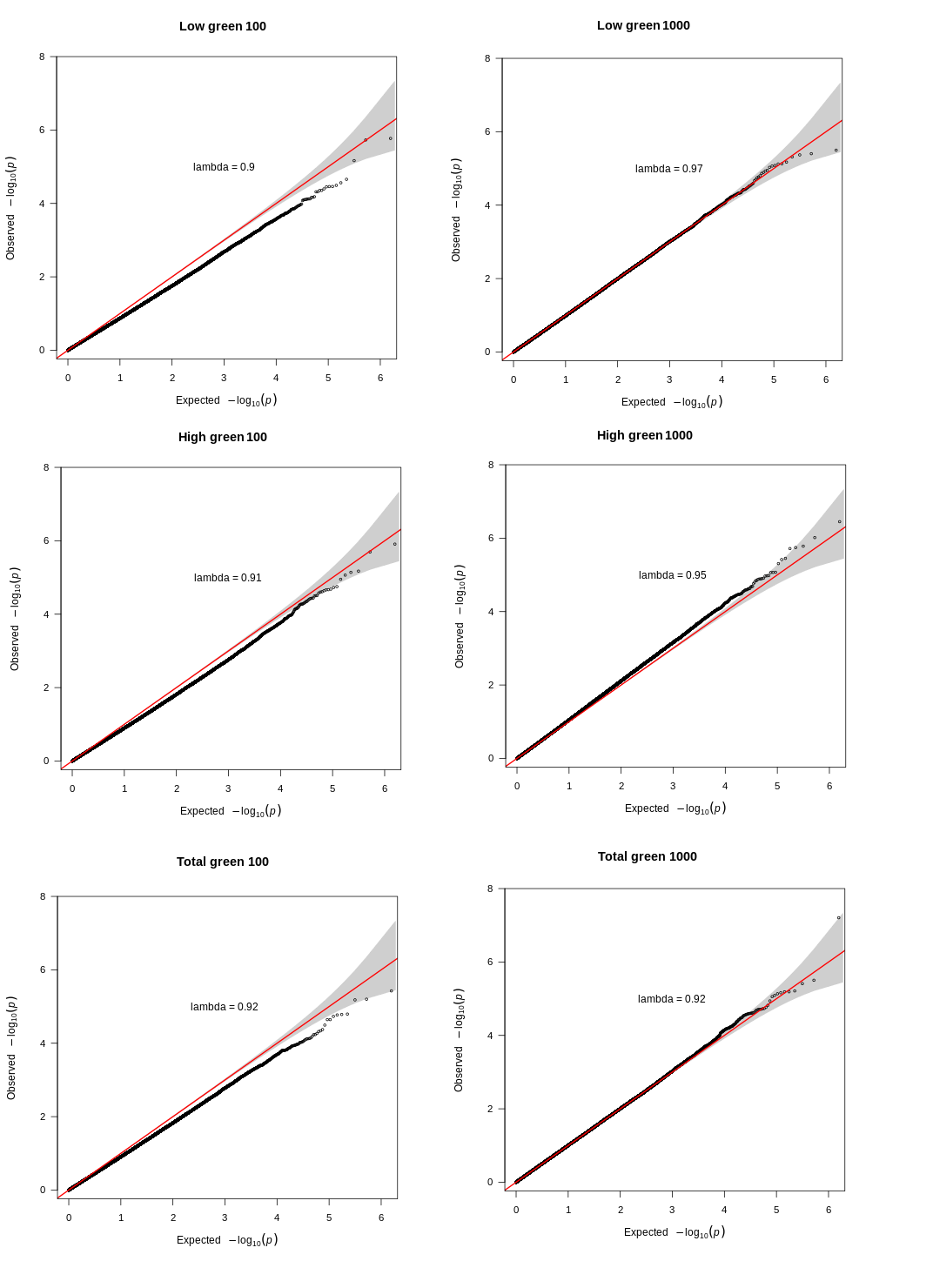


## Supplementary Figure 8

Precision plots comparing 1/median(standard error) to the square root of the mean sample size from single EWASs of maternal green space during pregnancy for children with 450K and EPIC methylation arrays.


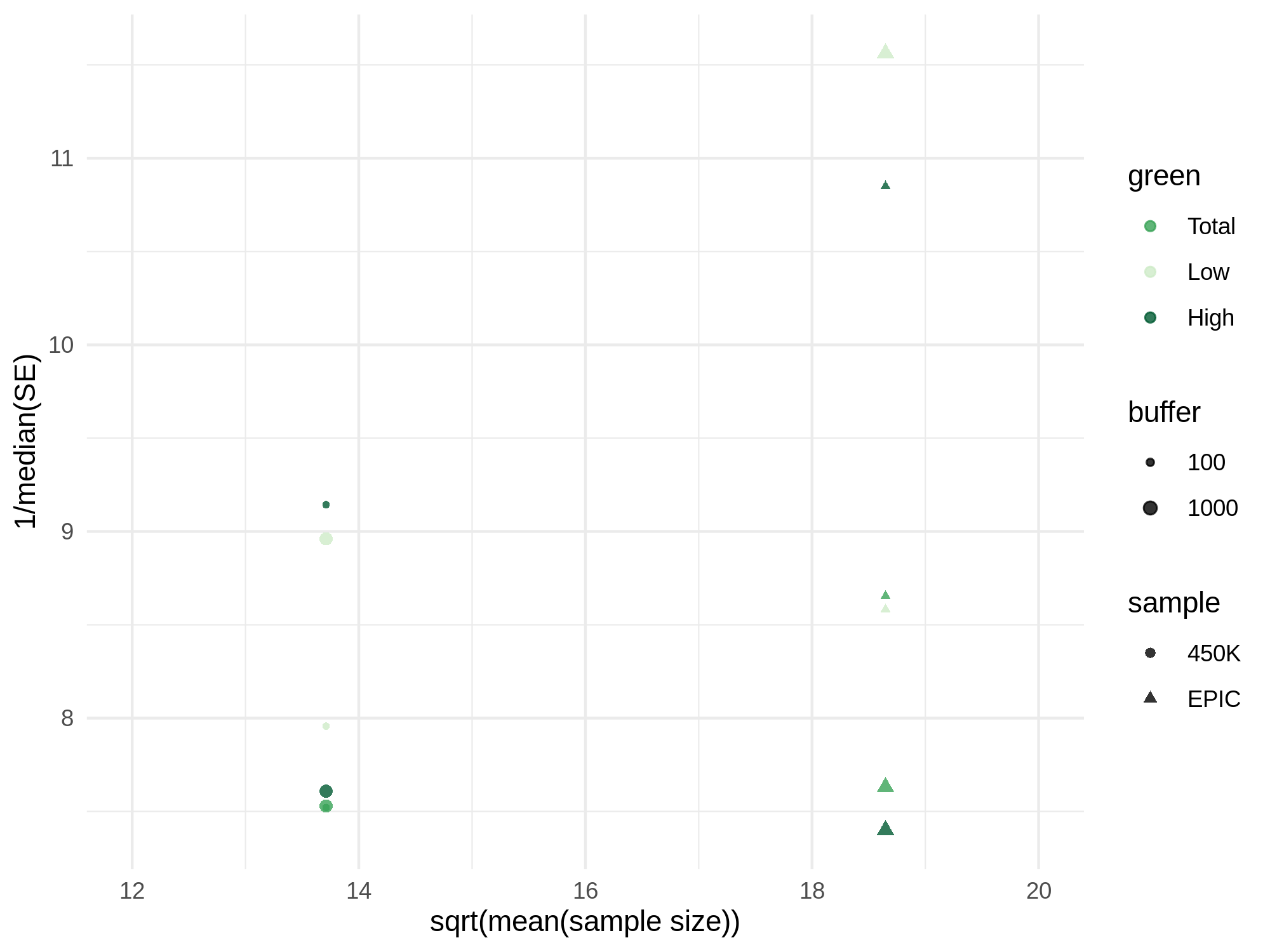


## Supplementary Figure 9

Scatter plots show the distribution of effect estimates from single EWASs of maternal green space during pregnancy for children with (A) 450K and (B) EPIC arrays methylation.


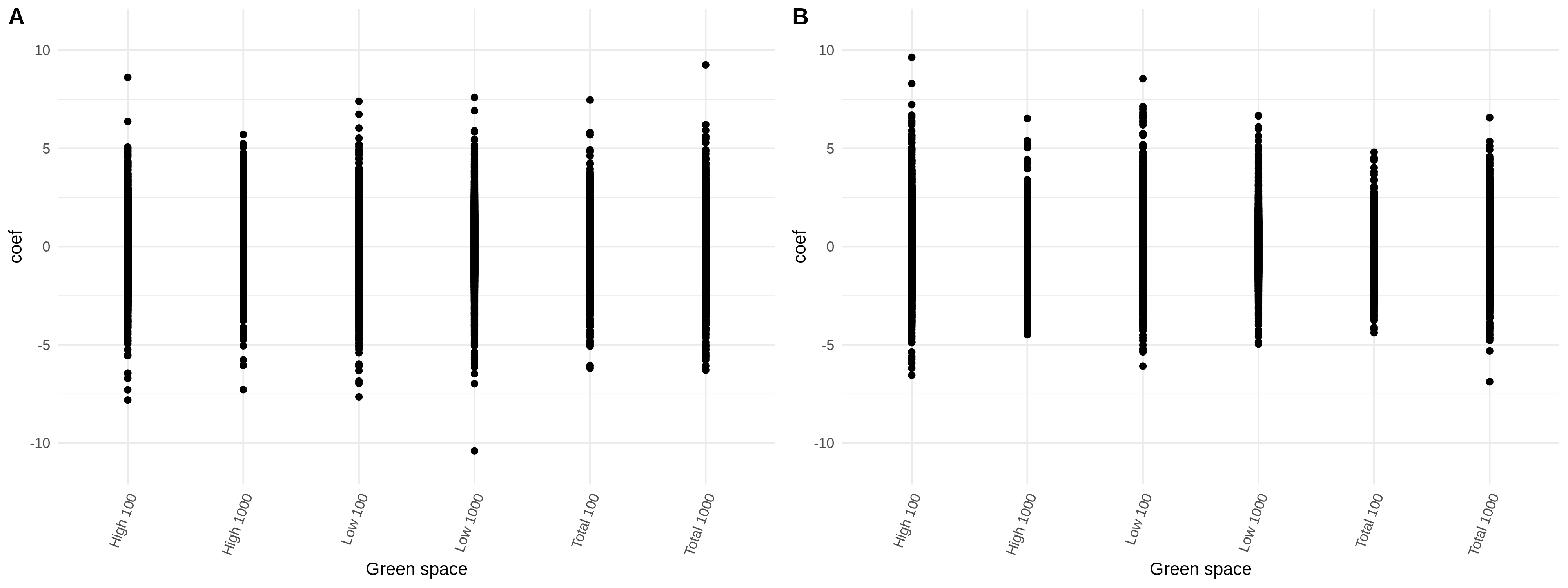


Coef= Estimates that represent % increase of DNA methylation per one interquartile range increase of exposure to maternal exposure to green space during pregnancy

## Supplementary Figure 10

Volcano plot shows estimates on x-axis and (-log10) p-values on y-axis for associations of the look-up analyses of CpGs annotated to seven HPA axis candidate genes (*NR3C1*, *FKBP5*, *11β-HSD2*, *CRH*, *CRHBP*, *SLC6A4*, and *OXT*) and maternal green space during pregnancy in meta-EWASs. No association had FDR corrected p-values <0.05. The black line represents the 0.05 p-value threshold.


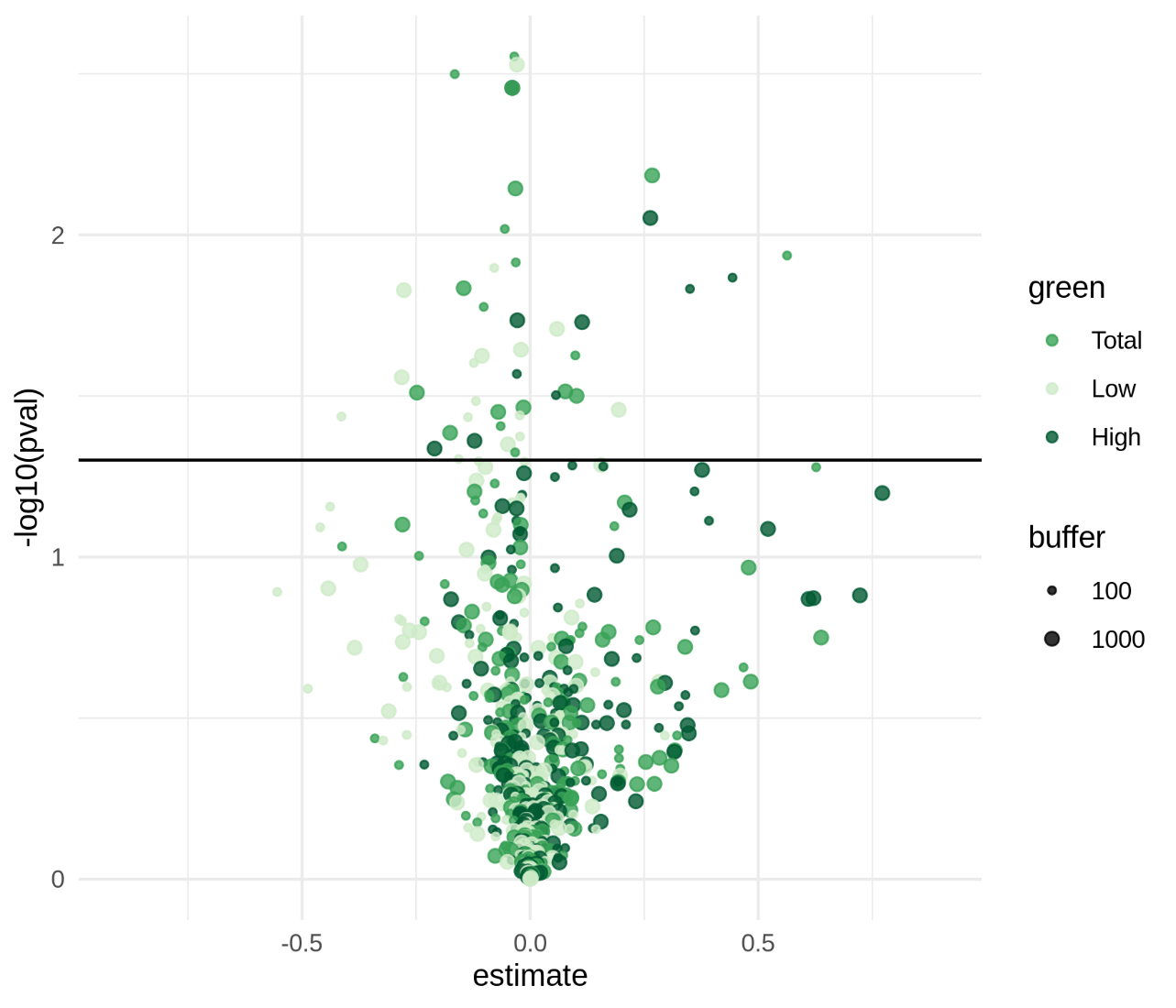


Estimates represent % increase of DNA methylation per one interquartile range increase of exposure to maternal exposure to green space during pregnancy

## Supplementary Figure 11

Volcano plot shows estimates on x-axis and (-log10) p-values on y-axis for associations of transcripts (corresponding to nearest genes annotated to differentially methylated regions associated with maternal green space during pregnancy in the meta-EWASs) and maternal green space during pregnancy in the 450K array samples. No association had FDR corrected p-values <0.05. The black line represents the 0.05 p-value threshold.


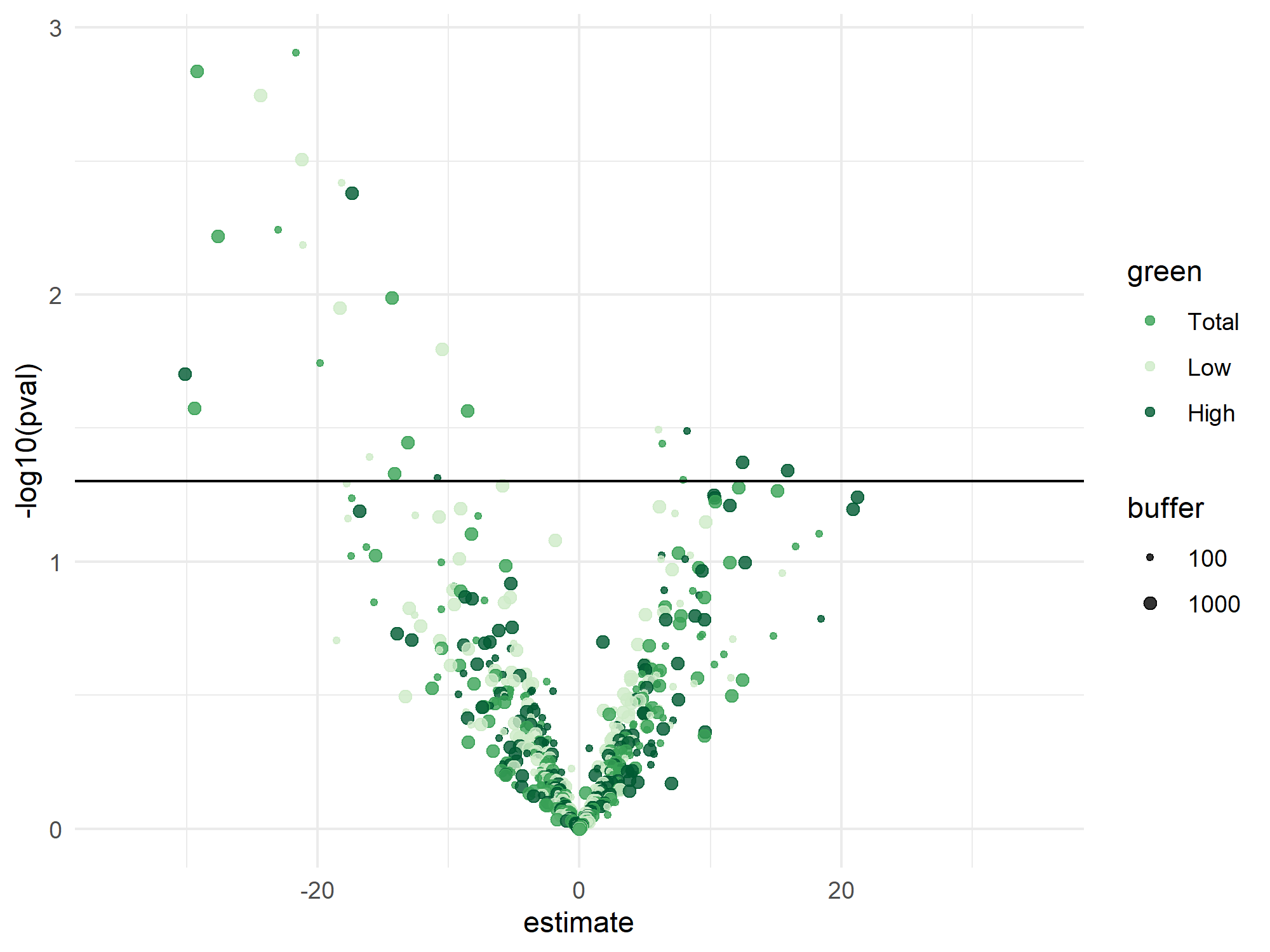


Estimates represent the log2 fold change of transcripts per one interquartile range increase of exposure to maternal exposure to green space during pregnancy

## Supplementary Figure 12

Venn diagram compares genes annotated to differentially methylated regions in our meta-EWASs with previous findings in the literature (by Jeong et^1^, Xu et al^2^, Lee et al^3^ and Dockx et al^4^).


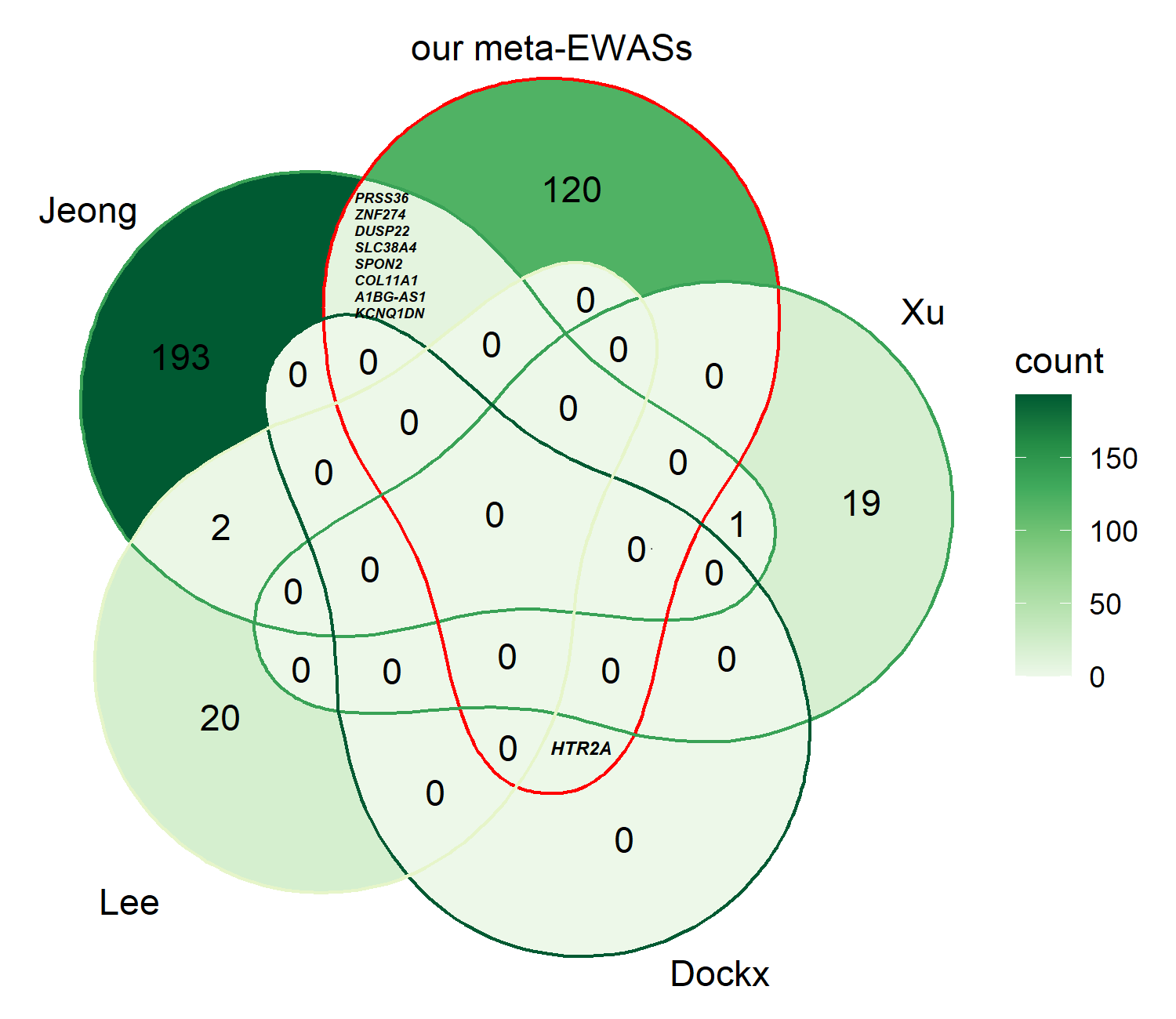


1. Jeong A, Eze IC, Vienneau D, et al. Residential greenness-related DNA methylation changes. Environ Int. 2022;158:106945.

2. Xu R, Li S, Li S, et al. Residential surrounding greenness and DNA methylation: An epigenome-wide association study. Environ Int. 2021;154:106556.

3. Lee KS, Choi YJ, Cho JW, et al. Children's Greenness Exposure and IQ-Associated DNA Methylation: A Prospective Cohort Study. Int J Environ Res Public Health. 2021;18(14):7429.

4. Dockx Y, Bijnens E, Saenen N, et al. Residential green space in association with the methylation status in a CpG site within the promoter region of the placental serotonin receptor HTR2A. Epigenetics. 2022:1-12.

## Supplementary Figure 13

(A) Venn diagram and (B) heatmap show the differentially methylated regions in main meta-EWASs and in the sensitivity analyses by adding as covariates to the models maternal exposure during pregnancy to PM_2.5_, distance to nearest major roads, urbanicity (coded as urban, suburban, and rural), and median neighborhood income (only regions identified by DMRcate are shown).


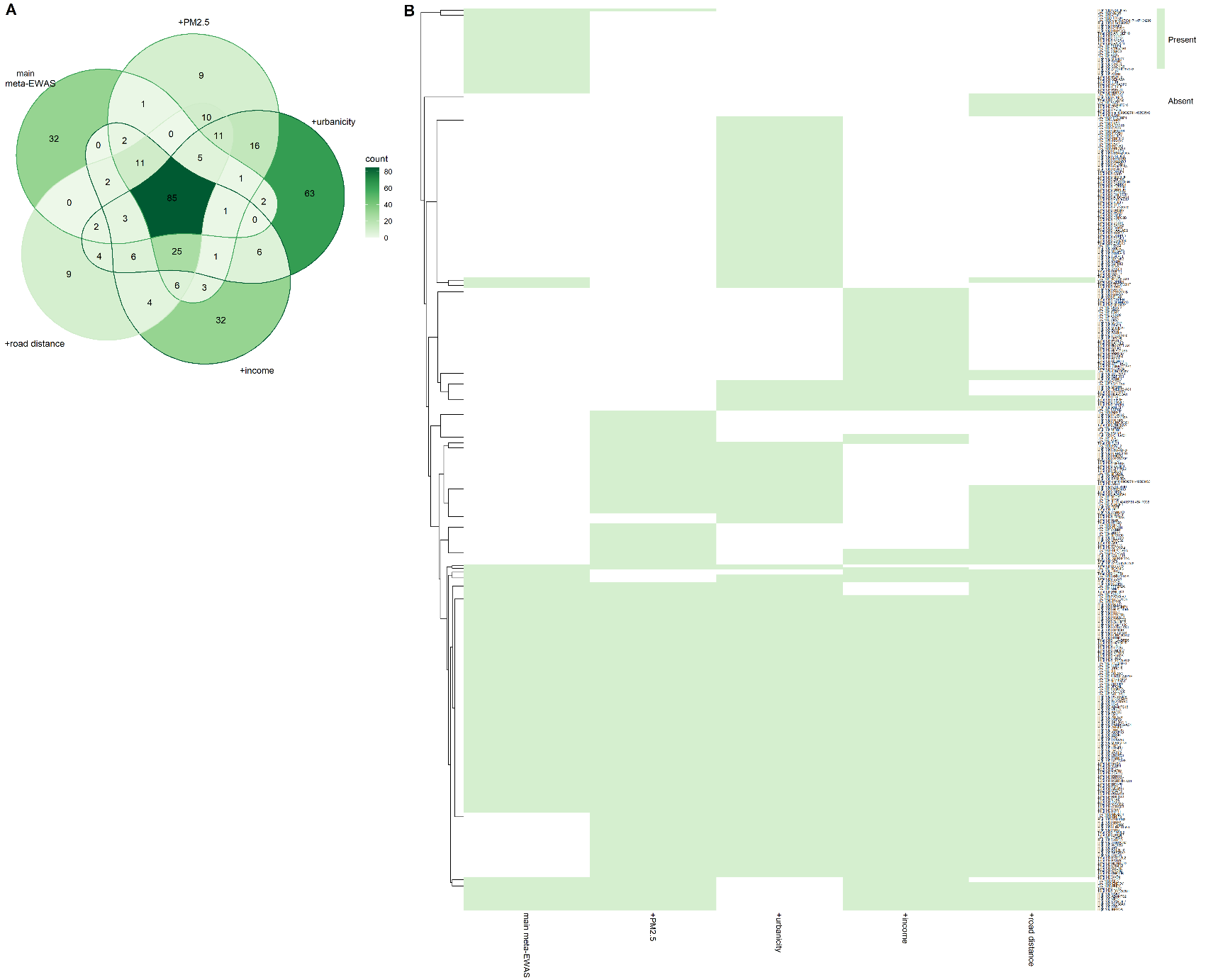


## Supplementary Figure 14

Heatmap shows the differentially methylated regions in main meta-EWASs and in the robustness analyses using non-trimmed DNA methylation.


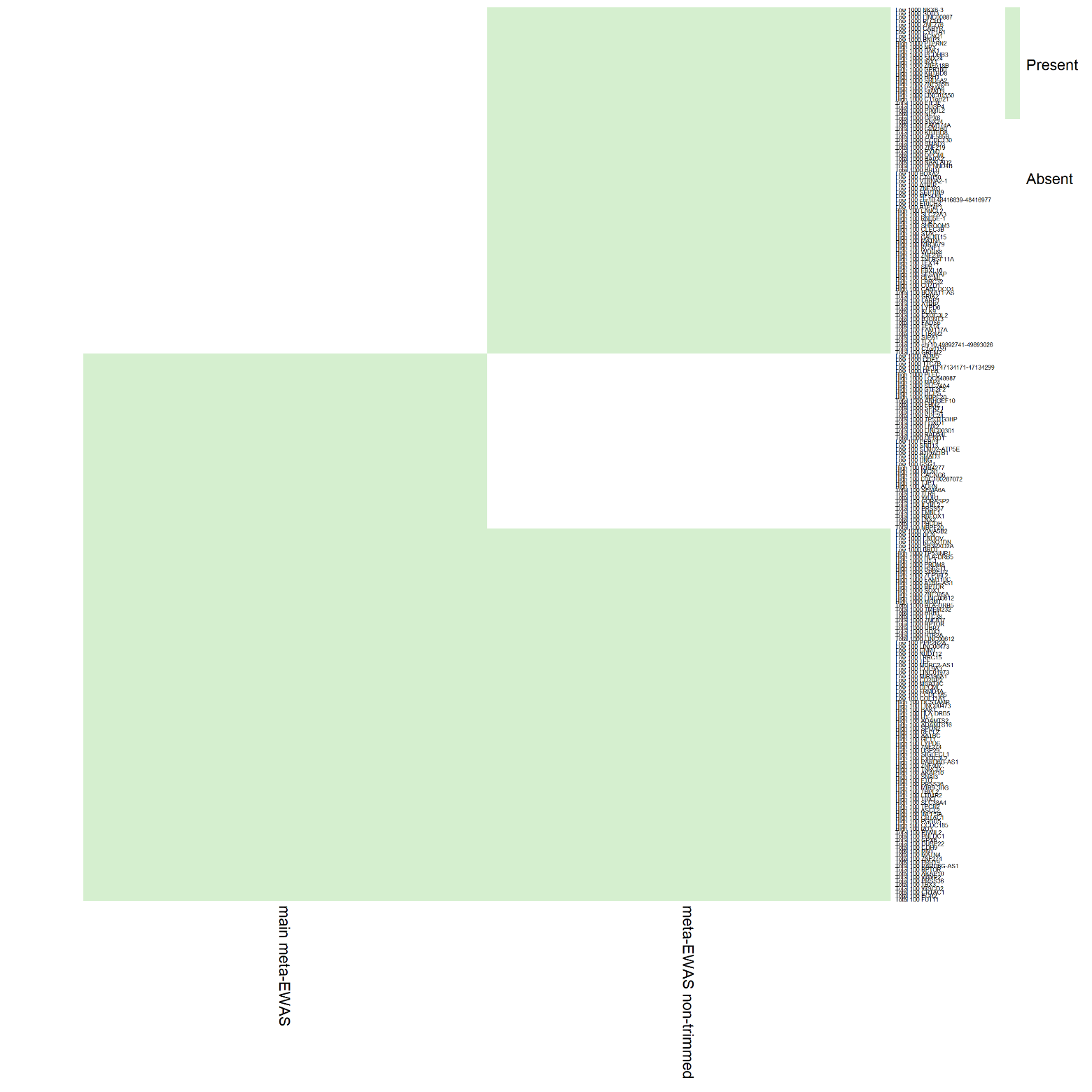


## Supplementary Table 1

Characteristics of the entire subsets and percentages (%) of missing data of the two ENVIR*ON*AGE subpopulations with 450K and EPIC DNA methylation data.

|  | **450K arrays samples (N=200)** | | **EPIC arrays samples**  **(N=377)** | |
| --- | --- | --- | --- | --- |
|  | **N/mean (%/sd)** | **Missing data %** | **N/mean (%/sd)** | **Missing data %** |
| Newborns’ sex, girls | 97 (48.74) | 0.5 | 194 (51.46) | 0 |
| Gestational age, weeks | 39.10 (1.64) | 0.5 | 39.13 (1.61) | 0 |
| Ethnicity, non-European | 22 (11.06) | 0.5 | 23 (6.10) | 0 |
| Maternal age, years | 29.36 (4.43) | 0.5 | 30.11 (4.24) | 0 |
| Parity, |  | 0.5 |  | 0 |
| *Primiparous* | 109 (54.77) |  | 205 (54.38) |  |
| *Secundiparous* | 60 (30.15) |  | 132 (35.01) |  |
| *Multiparous* | 30 (15.08) |  | 40 (10.61) |  |
| Season at delivery, |  | 0.5 |  | 0 |
| *Winter* | 46 (23.12) |  | 98 (25.99) |  |
| *Spring* | 84 (42.21) |  | 95 (25.20) |  |
| *Summer* | 27 (13.57) |  | 91 (24.14) |  |
| *Autumn* | 42 (21.11) |  | 93 (24.67) |  |
| Maternal smoking, yes | 26 (13.07) | 0.5 | 42 (11.17) | 0.3 |
| Maternal education, |  | 3 |  | 0 |
| *Low* | 27 (13.92) |  | 26 (6.90) |  |
| *Medium* | 67 (34.54) |  | 105 (27.85) |  |
| *High* | 100 (51.55) |  | 246 (65.25) |  |
| Green space |  |  |  | 0 |
| *Total 100m* | 0.47 (0.15) | 1.5 | 0.49 (0.15) | 0 |
| *Total 1000m* | 0.54 (0.15) | 1.5 | 0.53 (0.16) | 0 |
| *Low 100m* | 0.32 (0.11) | 1.5 | 0.33 (0.11) | 0 |
| *Low 1000m* | 0.24 (0.07) | 1.5 | 0.25 (0.07) | 0 |
| *High 100m* | 0.15 (0.13) | 1.5 | 0.16 (0.13) | 0 |
| *High 1000m* | 0.30 (0.14) | 1.5 | 0.28 (0.14) | 0 |
| DNA methylation |  |  |  | 0 |
| Samples N after QC and preprocessing | 197 (98.50) | 1.5 | 372 (98.7)* | 1.3 |

^* Please, note that 22 additional samples were remouved from the EPIC subset because they were already included in the 450K subset^

^N=number; sd=standard deviation^

## Supplementary Table 2

List of the 72 CpGs associated with green space in the meta-EWASs that were suggestive significant (p-values<1e-05).

| **Green** | **Buffer** | **CpG** | **Gene name** | **Gene Region** | **coef** | **se** | **p-value** | **I^2^** | **I^2^ p-value** |
| --- | --- | --- | --- | --- | --- | --- | --- | --- | --- |
| High | 1000 | cg14528046 | *PHF17* | Body | 0.48 | 0.09 | **5.68E-08** | 0.00 | 0.80 |
| Total | 100 | cg07269003 | *PAQR9* | 1stExon | 0.91 | 0.18 | 4.73E-07 | 0.00 | 0.67 |
| High | 1000 | cg00087735 | *SLC45A1* | Body | 0.48 | 0.10 | 6.21E-07 | 9.08 | 0.29 |
| Total | 1000 | cg05337024 | *TMEM159* | TSS200 | -0.05 | 0.01 | 7.36E-07 | 56.98 | 0.13 |
| Total | 1000 | cg04713272 |  |  | 0.39 | 0.08 | 9.24E-07 | 0.00 | 0.93 |
| Total | 1000 | cg19840493 |  |  | 0.08 | 0.02 | 1.40E-06 | 0.00 | 0.76 |
| Low | 1000 | cg17170743 | *PCGF3* | 5'UTR | -0.22 | 0.05 | 1.54E-06 | 39.14 | 0.20 |
| High | 1000 | cg08075631 | *C16orf58* | Body | 0.24 | 0.05 | 1.61E-06 | 0.00 | 0.92 |
| Total | 100 | cg15544004 |  |  | -0.53 | 0.11 | 1.66E-06 | 89.63 | 0.00 |
| Total | 100 | cg06099465 | *WDR81* | Body | -0.43 | 0.09 | 1.78E-06 | 0.00 | 0.87 |
| Low | 100 | cg03550233 | *KRT19* | TSS1500 | -0.62 | 0.13 | 1.96E-06 | 0.00 | 0.35 |
| High | 100 | cg10541043 | *CUX1* | Body | 0.19 | 0.04 | 2.15E-06 | 0.00 | 0.51 |
| Total | 100 | cg22720790 | *ZNF274* | Body | 2.08 | 0.44 | 2.19E-06 | 0.00 | 0.97 |
| High | 100 | cg08785784 |  |  | 1.14 | 0.24 | 2.28E-06 | 88.76 | 0.00 |
| Total | 100 | cg11647944 | *NTF3* | TSS200 | 0.31 | 0.06 | 2.30E-06 | 0.00 | 0.73 |
| Low | 1000 | cg05415931 | *SMOC1* | Body | -0.33 | 0.07 | 2.31E-06 | 0.00 | 0.60 |
| Total | 1000 | cg05000348 | *CDK2AP2* | TSS1500 | -0.16 | 0.03 | 2.75E-06 | 0.00 | 0.87 |
| Total | 1000 | cg05670865 | *TRIM27* | Body | -0.09 | 0.02 | 2.76E-06 | 0.00 | 0.40 |
| Total | 100 | cg00545705 | *PLXNA2* | Body | 0.50 | 0.11 | 2.84E-06 | 12.01 | 0.29 |
| Total | 1000 | cg00087735 | *SLC45A1* | Body | 0.44 | 0.09 | 3.06E-06 | 0.00 | 0.70 |
| Low | 100 | cg00854817 |  |  | -0.45 | 0.10 | 3.16E-06 | 0.00 | 0.58 |
| Total | 1000 | cg01532075 | *BFSP2* | Body | 0.50 | 0.11 | 3.18E-06 | 18.21 | 0.27 |
| High | 1000 | cg19651132 | *KCNA1* | TSS1500 | -0.05 | 0.01 | 3.24E-06 | 69.45 | 0.07 |
| Total | 1000 | cg22685369 | *SLC35F3* | TSS1500 | -0.06 | 0.01 | 3.69E-06 | 92.92 | 0.00 |
| Low | 100 | cg14543412 | *HIST1H3E* | TSS1500 | 0.13 | 0.03 | 3.72E-06 | 0.00 | 0.32 |
| Low | 100 | cg12644588 | *CAMTA1* | TSS200 | -0.11 | 0.02 | 3.74E-06 | 0.00 | 0.46 |
| High | 1000 | cg04483336 | *RPAIN* | Body | -0.07 | 0.02 | 4.02E-06 | 81.00 | 0.02 |
| High | 1000 | cg19976997 |  |  | 0.07 | 0.02 | 4.06E-06 | 40.59 | 0.19 |
| Total | 1000 | cg13291666 | *MAPK7* | TSS1500 | -0.78 | 0.17 | 4.14E-06 | 15.11 | 0.28 |
| High | 100 | cg23560756 | *PANX1* | TSS200 | -0.05 | 0.01 | 4.32E-06 | 0.00 | 0.54 |
| Low | 1000 | cg00080252 |  |  | -0.37 | 0.08 | 4.82E-06 | 69.24 | 0.07 |
| High | 1000 | cg13708759 | *SOHLH1* | 3'UTR | 0.75 | 0.16 | 5.00E-06 | 69.20 | 0.07 |
| Total | 1000 | cg26753208 |  |  | 1.64 | 0.36 | 5.15E-06 | 0.00 | 0.64 |
| Total | 1000 | cg13325584 | *XRCC6BP1* | TSS1500 | -0.12 | 0.03 | 5.62E-06 | 83.77 | 0.01 |
| High | 1000 | cg26807643 | *MAD2L2* | TSS1500 | -0.05 | 0.01 | 5.68E-06 | 75.02 | 0.05 |
| High | 1000 | cg19840493 |  |  | 0.07 | 0.02 | 5.70E-06 | 0.00 | 0.75 |
| Total | 1000 | cg00630583 | *PXMP2* | 1stExon | -0.06 | 0.01 | 5.78E-06 | 62.05 | 0.10 |
| High | 1000 | cg21027474 | *PGAM5* | Body | 0.12 | 0.03 | 5.83E-06 | 0.00 | 0.45 |
| Total | 100 | cg23440413 | *PCDHAC2* | 1stExon | -0.15 | 0.03 | 5.96E-06 | 0.00 | 0.99 |
| Low | 1000 | cg00770085 | *TTC39A* | TSS200 | -0.19 | 0.04 | 6.60E-06 | 0.00 | 0.64 |
| Total | 100 | cg09322259 |  |  | -0.04 | 0.01 | 6.64E-06 | 49.99 | 0.16 |
| High | 1000 | cg13505608 | *SLC34A3* | Body | 0.20 | 0.05 | 6.68E-06 | 0.00 | 0.73 |
| Total | 1000 | cg06205155 | *C15orf2* | TSS1500 | 0.40 | 0.09 | 6.70E-06 | 53.66 | 0.14 |
| Low | 1000 | cg01952793 | *RNF13* | TSS200 | -0.14 | 0.03 | 6.77E-06 | 0.00 | 0.56 |
| High | 1000 | cg00349948 |  |  | 0.52 | 0.11 | 6.87E-06 | 41.67 | 0.19 |
| Low | 100 | cg25277509 | *FGF9* | Body | -0.07 | 0.02 | 6.93E-06 | 0.00 | 0.38 |
| Total | 100 | cg18286862 | *ADAD1* | 5'UTR | 0.13 | 0.03 | 6.94E-06 | 4.44 | 0.31 |
| High | 100 | cg14772955 | *KIAA0319L* | TSS1500 | 0.48 | 0.11 | 6.98E-06 | 0.00 | 0.51 |
| Total | 1000 | cg02667487 | *NRN1L* | Body | 0.05 | 0.01 | 7.32E-06 | 8.57 | 0.30 |
| Low | 100 | cg07585502 |  |  | 0.32 | 0.07 | 7.41E-06 | 0.00 | 0.82 |
| Total | 1000 | cg14528046 | *PHF17* | Body | 0.40 | 0.09 | 7.51E-06 | 0.00 | 0.86 |
| Total | 1000 | cg00866215 |  |  | 0.39 | 0.09 | 7.56E-06 | 0.00 | 0.39 |
| High | 1000 | cg00230924 |  |  | 0.50 | 0.11 | 7.64E-06 | 0.00 | 0.41 |
| Total | 100 | cg13731338 |  |  | -0.10 | 0.02 | 7.64E-06 | 82.46 | 0.02 |
| High | 1000 | cg01532075 | *BFSP2* | Body | 0.49 | 0.11 | 7.65E-06 | 31.54 | 0.23 |
| Low | 1000 | cg13296579 | *OPCML* | Body | 1.11 | 0.25 | 8.15E-06 | 0.00 | 0.34 |
| Low | 100 | cg00361696 | *ANKHD1* | TSS1500 | -0.20 | 0.04 | 8.22E-06 | 0.00 | 0.79 |
| Total | 100 | cg03208951 | *EMP3* | 1stExon | -0.07 | 0.01 | 8.30E-06 | 59.03 | 0.12 |
| Low | 100 | cg18126320 | *CAPN9* | Body | -2.40 | 0.54 | 8.34E-06 | 23.91 | 0.25 |
| High | 1000 | cg11881861 | *SGSM2* | Body | 0.47 | 0.11 | 8.52E-06 | 0.00 | 0.69 |
| Total | 100 | cg06837568 | *WSCD2* | Body | 2.36 | 0.53 | 8.55E-06 | 32.15 | 0.22 |
| Low | 1000 | cg05635940 | *LGI4* | Body | -0.34 | 0.08 | 8.56E-06 | 0.00 | 0.76 |
| Total | 1000 | cg18435820 | *SPRY1* | 5'UTR | -0.04 | 0.01 | 8.85E-06 | 48.34 | 0.16 |
| Total | 1000 | cg05533744 | *E2F4* | Body | -0.09 | 0.02 | 9.01E-06 | 92.68 | 0.00 |
| Total | 1000 | cg01939414 | *MFSD9* | TSS1500 | -0.12 | 0.03 | 9.05E-06 | 0.00 | 0.59 |
| High | 1000 | cg18435820 | *SPRY1* | 5'UTR | -0.04 | 0.01 | 9.37E-06 | 55.15 | 0.14 |
| Low | 1000 | cg08736072 | *C16orf91* | Body | 0.56 | 0.13 | 9.37E-06 | 0.00 | 0.64 |
| High | 1000 | cg13291666 | *MAPK7* | TSS1500 | -0.76 | 0.17 | 9.41E-06 | 0.00 | 0.54 |
| Total | 1000 | cg04321378 | *SF3B2* | TSS1500 | -0.10 | 0.02 | 9.44E-06 | 67.86 | 0.08 |
| Low | 100 | cg24398933 | *CGN* | Body | 0.43 | 0.10 | 9.66E-06 | 0.00 | 0.75 |
| Total | 1000 | cg07346343 | *MED12L* | 1stExon | -0.20 | 0.05 | 9.85E-06 | 81.54 | 0.02 |
| Total | 100 | cg14239592 | *ZNF274* | Body | 1.03 | 0.23 | 9.92E-06 | 0.00 | 0.64 |

P-value in bold is genome-wide significant. Result are coloured in red in case of high heterogeneity (I^2^ >50%). coef=% increase of DNA methylation per one interquartile range increase of maternal exposure to green space during pregnancy; se= standard error, I^2^= heterogeneity

## Supplementary Table 3

Number of CpGs having I_2_>50 in the meta-EWASs of maternal exposure to green space during pregnancy.

| **Meta-EWASs of maternal pregnancy exposure to green space** | **Number of CpGs with I_2_>50** |
| --- | --- |
| *Total 100m* | 84,625 |
| *Total 1000m* | 94,876 |
| *Low 100m* | 67,757 |
| *Low 1000m* | 85,084 |
| *High 100m* | 67,626 |
| *High 1000m* | 85,956 |

I_2_= heterogeneity

## Supplementary Table 4

Complete list of the differentially methylated regions associated with maternal residential green space exposure during the pregnancy in children in the meta-EWASs.

| **DMRcate** | | | | | | | **ENmix-combp** | | | | | | |
| --- | --- | --- | --- | --- | --- | --- | --- | --- | --- | --- | --- | --- | --- |
| **Green space** | **Buffer** | **Genomic location** | **N** | **Nearest Gene** | **FDR** | **β** | **Green space** | **Buffer** | **Genomic location** | **N** | **Nearest Gene** | **Siddak- P value** | **β** |
| Total green | 100 | chr1:120255941-120255992 | 2 | *PHGDH* | 3.97E-03 | 0.80 | Total green | 100 | chr1:120255940-120255992 | 2 | *PHGDH* | 7.95E-06 | 0.80 |
| Total green | 100 | chr1:145384929-145385758 | 4 | *NBPF20* | 8.77E-05 | 1.11 | Total green | 100 | chr1:145385390-145385677 | 2 | *NBPF20* | 1.04E-05 | 1.75 |
| Total green | 100 | chr10:64572581-64573476 | 5 | *EGR2* | 1.19E-03 | 0.43 | Total green | 100 | chr10:64573183-64573476 | 3 | *EGR2* | 2.82E-05 | 0.45 |
| Total green | 100 | chr10:75533025-75533431 | 5 | *FUT11* | 3.97E-03 | -0.84 | Total green | 100 | chr10:75533138-75533431 | 4 | *FUT11* | 6.57E-06 | -1.03 |
| Total green | 100 | chr10:99734081-99735133 | 8 | *CRTAC1* | 1.97E-03 | 1.14 | Total green | 100 | chr10:99734661-99734912 | 3 | *CRTAC1* | 5.77E-05 | 1.45 |
| Total green | 100 | chr12:108634147-108634275 | 3 | *WSCD2* | 1.88E-05 | 1.52 | Total green | 100 | chr12:108634146-108634275 | 3 | *WSCD2* | 8.34E-09 | 1.52 |
| Total green | 100 | chr12:115112189-115113212 | 5 | *TBX3* | 2.74E-04 | 0.73 | Total green | 100 | chr12:115112188-115112626 | 4 | *TBX3* | 3.73E-06 | 0.80 |
| Total green | 100 | chr13:28194706-28194884 | 6 | *LNX2* | 1.10E-02 | -0.05 | Total green | 100 | chr13:28194705-28194884 | 7 | *LNX2* | 7.17E-06 | 0.04 |
| Total green | 100 | chr16:7703812-7703947 | 3 | *RBFOX1* | 5.97E-03 | 0.82 | Total green | 100 | chr16:7703811-7703947 | 4 | *RBFOX1* | 7.95E-06 | 0.63 |
| Total green | 100 | chr16:31159558-31159920 | 5 | *PRSS36* | 3.41E-04 | 0.85 | Total green | 100 | chr16:31159557-31159920 | 7 | *PRSS36* | 2.80E-07 | 0.59 |
| Total green | 100 | chr16:69966641-69967212 | 7 | *WWP2* | 2.17E-04 | 1.01 | Total green | 100 | chr16:69966814-69967212 | 6 | *WWP2* | 2.53E-07 | 1.16 |
| Total green | 100 | chr17:19883326-19883716 | 4 | *AKAP10* | 2.18E-03 | 0.81 | Total green | 100 | chr17:19883325-19883716 | 5 | *AKAP10* | 6.09E-06 | 0.66 |
| Total green | 100 | chr17:43318610-43318760 | 3 | *FMNL1* | 7.53E-03 | 0.08 | Total green | 100 | chr17:43318609-43318760 | 3 | *FMNL1* | 7.95E-06 | 0.08 |
| Total green | 100 | chr17:78864088-78865755 | 8 | *RPTOR* | 2.43E-03 | 0.84 | Total green | 100 | chr17:78865086-78865662 | 6 | *RPTOR* | 3.50E-06 | 0.79 |
| Total green | 100 | chr18:77905119-77905947 | 8 | *PARD6G-AS1* | 5.19E-05 | 1.22 | Total green | 100 | chr18:77905390-77905947 | 8 | *PARD6G-AS1* | 2.88E-08 | 1.12 |
| Total green | 100 | chr19:695447-695642 | 4 | *PRSS57* | 1.60E-02 | -0.27 | Total green | 100 | chr19:695446-695642 | 8 | *PRSS57* | 1.31E-05 | -0.14 |
| Total green | 100 | chr19:42811017-42811090 | 3 | *PRR19* | 4.43E-02 | 0.74 | Total green | 100 | chr19:42811016-42811173 | 4 | *PRR19* | 1.91E-05 | 0.65 |
| Total green | 100 | chr19:58715251-58716517 | 8 | *ZNF274* | 6.40E-14 | 1.07 | Total green | 100 | chr19:58715250-58716135 | 12 | *ZNF274* | 2.39E-16 | 0.65 |
| Total green | 100 | chr2:102867420-102867466 | 2 | *IL1RL2* | 1.56E-02 | 0.62 | Total green | 100 | chr2:102867419-102867466 | 2 | *IL1RL2* | 1.07E-05 | 0.62 |
| Total green | 100 | chr2:171784674-171785547 | 6 | *GORASP2* | 4.14E-04 | 0.31 | Total green | 100 | chr2:171785026-171785124 | 2 | *GORASP2* | 7.95E-06 | 0.83 |
| Total green | 100 | chr20:43935222-43935551 | 10 | *MATN4* | 5.17E-04 | 0.69 | Total green | 100 | chr20:43935221-43935551 | 11 | *MATN4* | 3.81E-07 | 0.63 |
| Total green | 100 | chr22:28073992-28074146 | 4 | *MN1* | 2.59E-02 | 1.09 | Total green | 100 | chr22:28073991-28074146 | 4 | *MN1* | 1.04E-05 | 1.09 |
| Total green | 100 | chr4:10118459-10118935 | 4 | *WDR1* | 8.54E-03 | -0.04 | Total green | 100 | chr4:10118458-10118677 | 3 | *WDR1* | 1.07E-05 | -0.03 |
| Total green | 100 | chr4:38858536-38858561 | 2 | *TLR6* | 3.32E-02 | 0.71 | Total green | 100 | chr4:38858535-38858561 | 3 | *TLR6* | 2.82E-05 | 0.45 |
| Total green | 100 | chr5:27038605-27038836 | 5 | *CDH9* | 4.36E-02 | -1.00 | Total green | 100 | chr5:27038604-27038836 | 5 | *CDH9* | 2.82E-05 | -1.00 |
| Total green | 100 | chr5:115910592-115910729 | 2 | *SEMA6A* | 2.52E-02 | -0.09 | Total green | 100 | chr5:115910591-115910729 | 2 | *SEMA6A* | 4.03E-05 | -0.09 |
| Total green | 100 | chr6:292385-293331 | 6 | *DUSP22* | 1.87E-03 | -2.51 | Total green | 100 | chr6:292521-292823 | 4 | *DUSP22* | 2.82E-05 | -2.01 |
| Total green | 100 | chr6:28973318-28973828 | 11 | *GPX6* | 6.17E-03 | -0.11 | Total green | 100 | chr6:28973317-28973524 | 10 | *GPX6* | 7.17E-06 | -0.11 |
| Total green | 100 | chr6:160240600-160241556 | 5 | *PNLDC1* | 2.55E-04 | -1.57 | Total green | 100 | chr6:160241104-160241556 | 4 | *PNLDC1* | 3.81E-07 | -1.85 |
| Total green | 100 | chr8:22132714-22133076 | 8 | *PIWIL2* | 2.59E-02 | 1.10 | Total green | 100 | chr8:22132991-22133076 | 3 | *PIWIL2* | 9.38E-04 | 1.18 |
| High green | 100 | chr1:979484-979589 | 2 | *AGRN* | 1.24E-02 | 0.21 | High green | 100 | chr1:979483-979589 | 5 | *AGRN* | 3.69E-05 | 0.07 |
| High green | 100 | chr1:211652276-211652688 | 3 | *RD3* | 2.43E-03 | 1.03 | High green | 100 | chr1:211652275-211652549 | 2 | *RD3* | 3.63E-05 | 0.84 |
| High green | 100 | chr1:223566127-223567002 | 9 | *CCDC185* | 1.87E-08 | 0.83 | High green | 100 | chr1:223566446-223567002 | 7 | *CCDC185* | 1.17E-10 | 0.96 |
| High green | 100 | chr1:230468464-230468773 | 5 | *PGBD5* | 4.38E-03 | 0.76 | High green | 100 | chr1:230468463-230468773 | 5 | *PGBD5* | 3.24E-06 | 0.76 |
| High green | 100 | chr10:99734081-99735202 | 9 | *CRTAC1* | 5.05E-08 | 1.14 | High green | 100 | chr10:99734080-99735202 | 10 | *CRTAC1* | 8.80E-12 | 1.02 |
| High green | 100 | chr10:134332277-134332442 | 3 | *INPP5A* | 4.25E-02 | 0.91 | High green | 100 | chr10:134332276-134332442 | 3 | *INPP5A* | 3.55E-05 | 0.91 |
| High green | 100 | chr11:2292544-2293665 | 22 | *ASCL2* | 1.52E-05 | 0.40 | High green | 100 | chr11:2292889-2293305 | 17 | *ASCL2* | 1.49E-07 | 0.50 |
| High green | 100 | chr11:68924577-68925191 | 6 | *TPCN2* | 7.22E-04 | 0.66 | High green | 100 | chr11:68924576-68924956 | 5 | *TPCN2* | 2.60E-06 | 0.76 |
| High green | 100 | chr12:47225979-47226453 | 6 | *SLC38A4* | 2.89E-02 | 0.67 | High green | 100 | chr12:47226004-47226255 | 3 | *SLC38A4* | 2.15E-03 | 0.76 |
| High green | 100 | chr12:115112189-115113212 | 5 | *TBX3* | 1.45E-03 | 0.56 | High green | 100 | chr12:115112432-115112626 | 2 | *TBX3* | 3.30E-05 | 0.81 |
| High green | 100 | chr14:24779793-24780926 | 13 | *LTB4R2* | 2.83E-06 | 0.74 | High green | 100 | chr14:24780403-24780926 | 10 | *LTB4R2* | 1.58E-07 | 0.73 |
| High green | 100 | chr14:55907007-55907501 | 8 | *TBPL2* | 3.44E-03 | 0.58 | High green | 100 | chr14:55907373-55907501 | 6 | *TBPL2* | 3.24E-06 | 0.70 |
| High green | 100 | chr15:29968032-29968124 | 2 | *TJP1* | 2.02E-03 | 1.30 | High green | 100 | chr15:29968031-29968124 | 2 | *TJP1* | 1.49E-05 | 1.30 |
| High green | 100 | chr15:89959984-89960743 | 7 | *MIR9-3HG* | 6.55E-05 | 0.79 | High green | 100 | chr15:89960277-89960743 | 6 | *MIR9-3HG* | 3.63E-07 | 0.74 |
| High green | 100 | chr16:31159558-31160393 | 6 | *PRSS36* | 4.93E-05 | 0.60 | High green | 100 | chr16:31159557-31159920 | 6 | *PRSS36* | 4.59E-08 | 0.61 |
| High green | 100 | chr16:54227790-54228582 | 7 | *FTO* | 4.26E-06 | 0.71 | High green | 100 | chr16:54227789-54228582 | 7 | *FTO* | 8.87E-10 | 0.71 |
| High green | 100 | chr16:88744522-88744842 | 3 | *SNAI3* | 2.98E-03 | 0.20 | High green | 100 | chr16:88744521-88744681 | 2 | *SNAI3* | 1.51E-05 | 0.27 |
| High green | 100 | chr17:19883326-19883716 | 4 | *AKAP10* | 2.73E-02 | 0.57 | High green | 100 | chr17:19883325-19883602 | 4 | *AKAP10* | 4.49E-05 | 0.50 |
| High green | 100 | chr17:20687481-20687681 | 2 | *LOC100287072* | 1.88E-02 | 0.88 | High green | 100 | chr17:20687480-20687681 | 2 | *LOC100287072* | 3.73E-05 | 0.88 |
| High green | 100 | chr17:76037035-76037562 | 6 | *TNRC6C* | 8.86E-03 | 0.61 | High green | 100 | chr17:76037034-76037250 | 5 | *TNRC6C* | 3.73E-05 | 0.53 |
| High green | 100 | chr18:72837386-72837782 | 6 | *ZNF407* | 4.31E-03 | 0.81 | High green | 100 | chr18:72837530-72837782 | 5 | *ZNF407* | 4.86E-06 | 0.86 |
| High green | 100 | chr18:77905119-77905947 | 9 | *PARD6G-AS1* | 5.06E-07 | 1.12 | High green | 100 | chr18:77905118-77905947 | 11 | *PARD6G-AS1* | 3.55E-10 | 0.96 |
| High green | 100 | chr19:45737208-45737880 | 9 | *EXOC3L2* | 1.16E-02 | 0.42 | High green | 100 | chr19:45737482-45737880 | 12 | *EXOC3L2* | 2.24E-05 | 0.29 |
| High green | 100 | chr19:51774377-51774666 | 5 | *SIGLECL1* | 2.18E-02 | 0.63 | High green | 100 | chr19:51774376-51774666 | 5 | *SIGLECL1* | 2.42E-05 | 0.63 |
| High green | 100 | chr19:54496353-54496611 | 2 | *CACNG6* | 1.80E-02 | 0.80 | High green | 100 | chr19:54496352-54496611 | 2 | *CACNG6* | 4.20E-05 | 0.80 |
| High green | 100 | chr19:57630202-57630742 | 13 | *USP29* | 7.40E-03 | 0.38 | High green | 100 | chr19:57630535-57630742 | 8 | *USP29* | 5.96E-05 | 0.41 |
| High green | 100 | chr19:58715251-58716135 | 6 | *ZNF274* | 3.90E-07 | 0.73 | High green | 100 | chr19:58715350-58716135 | 10 | *ZNF274* | 2.83E-09 | 0.36 |
| High green | 100 | chr2:150176791-150177277 | 3 | *LYPD6* | 7.10E-03 | 0.87 | High green | 100 | chr2:150176790-150177004 | 2 | *LYPD6* | 3.67E-05 | 0.77 |
| High green | 100 | chr21:40759534-40760258 | 6 | *GET1* | 1.26E-03 | 0.89 | High green | 100 | chr21:40759533-40759694 | 5 | *GET1* | 6.79E-06 | 0.89 |
| High green | 100 | chr21:45246308-45246441 | 3 | *AATBC* | 9.70E-03 | 0.85 | High green | 100 | chr21:45246307-45246441 | 3 | *AATBC* | 8.51E-06 | 0.85 |
| High green | 100 | chr22:32599511-32599648 | 4 | *RFPL2* | 5.02E-03 | 1.68 | High green | 100 | chr22:32599510-32599648 | 4 | *RFPL2* | 5.23E-06 | 1.68 |
| High green | 100 | chr3:49459855-49460177 | 8 | *NICN1* | 1.21E-02 | 0.36 | High green | 100 | chr3:49459854-49460162 | 8 | *NICN1* | 1.76E-05 | 0.34 |
| High green | 100 | chr4:1166528-1166990 | 9 | *SPON2* | 8.66E-03 | 0.25 | High green | 100 | chr4:1166766-1166990 | 11 | *SPON2* | 1.67E-05 | 0.20 |
| High green | 100 | chr5:1724892-1725823 | 4 | *MIR4277* | 6.14E-05 | 0.78 | High green | 100 | chr5:1725284-1725411 | 2 | *MIR4277* | 5.10E-06 | 1.09 |
| High green | 100 | chr5:5139069-5139643 | 7 | *ADAMTS16* | 8.30E-03 | 0.51 | High green | 100 | chr5:5139457-5139512 | 4 | *ADAMTS16* | 9.20E-03 | 0.47 |
| High green | 100 | chr5:178547863-178548373 | 3 | *ADAMTS2* | 4.30E-03 | 1.42 | High green | 100 | chr5:178548228-178548373 | 2 | *ADAMTS2* | 3.75E-05 | 1.53 |
| High green | 100 | chr6:26017619-26018534 | 8 | *H1-1* | 8.66E-04 | 0.35 | High green | 100 | chr6:26017938-26018127 | 6 | *H1-1* | 9.27E-06 | 0.34 |
| High green | 100 | chr6:32063394-32063991 | 15 | *HLA-DRB5* | 6.13E-03 | 0.72 | High green | 100 | chr6:32063606-32063901 | 9 | *HLA-DRB5* | 1.75E-04 | 0.89 |
| High green | 100 | chr6:33091111-33092097 | 10 | *BAK1* | 1.69E-03 | 0.29 | High green | 100 | chr6:33091566-33091841 | 5 | *BAK1* | 4.86E-06 | 0.49 |
| High green | 100 | chr6:166232637-166233141 | 4 | *LINC00473* | 2.36E-03 | 0.66 | High green | 100 | chr6:166232959-166233141 | 3 | *LINC00473* | 2.17E-06 | 0.83 |
| High green | 100 | chr8:105379368-105379985 | 6 | *DCSTAMP* | 2.11E-06 | 0.62 | High green | 100 | chr8:105379367-105379726 | 5 | *DCSTAMP* | 7.09E-09 | 0.57 |
| Low green | 100 | chr1:103574469-103574619 | 4 | *COL11A1* | 2.43E-02 | -0.62 | Low green | 100 | chr1:103574468-103574619 | 4 | *COL11A1* | 9.64E-06 | -0.62 |
| Low green | 100 | chr1:223566127-223567002 | 8 | *CCDC185* | 3.15E-04 | -0.95 | Low green | 100 | chr1:223566642-223566794 | 5 | *CCDC185* | 3.55E-06 | -1.07 |
| Low green | 100 | chr10:14051636-14052028 | 6 | *FRMD4A* | 7.86E-07 | -1.29 | Low green | 100 | chr10:14051635-14052028 | 7 | *FRMD4A* | 7.23E-10 | -1.15 |
| Low green | 100 | chr11:133098424-133098499 | 3 | *OPCML* | 1.76E-02 | -0.39 | Low green | 100 | chr11:133098423-133098499 | 4 | *OPCML* | 1.97E-05 | -0.26 |
| Low green | 100 | chr12:13254620-13254673 | 2 | *GSG1* | 3.47E-03 | -0.17 | Low green | 100 | chr12:13254619-13254673 | 2 | *GSG1* | 7.48E-06 | -0.17 |
| Low green | 100 | chr12:86658745-86659008 | 3 | *MGAT4C* | 3.72E-02 | -0.92 | Low green | 100 | chr12:86658744-86659008 | 3 | *MGAT4C* | 2.01E-05 | -0.92 |
| Low green | 100 | chr12:109535838-109536430 | 6 | *UNG* | 1.95E-03 | -0.02 | Low green | 100 | chr12:109535837-109536106 | 4 | *UNG* | 3.30E-05 | -0.01 |
| Low green | 100 | chr15:67355497-67356942 | 5 | *SMAD3* | 2.58E-05 | 2.33 | Low green | 100 | chr15:67356309-67356387 | 2 | *SMAD3* | 4.65E-06 | 2.71 |
| Low green | 100 | chr16:30366293-30367227 | 11 | *CD2BP2* | 5.00E-03 | -0.06 | Low green | 100 | chr16:30366664-30366847 | 6 | *CD2BP2* | 1.63E-04 | -0.08 |
| Low green | 100 | chr17:46719276-46720050 | 4 | *MIR196A1* | 5.46E-04 | -0.40 | Low green | 100 | chr17:46719275-46719761 | 5 | *MIR196A1* | 2.80E-06 | -0.26 |
| Low green | 100 | chr17:75789279-75789439 | 4 | *LINC01973* | 3.72E-02 | -0.82 | Low green | 100 | chr17:75789278-75789439 | 4 | *LINC01973* | 2.27E-05 | -0.82 |
| Low green | 100 | chr2:71192118-71192262 | 2 | *ATP6V1B1* | 1.42E-02 | -0.15 | Low green | 100 | chr2:71192117-71192262 | 2 | *ATP6V1B1* | 1.83E-05 | -0.15 |
| Low green | 100 | chr20:57607406-57607692 | 7 | *SLMO2-ATP5E* | 3.51E-02 | -0.04 | Low green | 100 | chr20:57607405-57607440 | 4 | *SLMO2-ATP5E* | 8.03E-04 | -0.04 |
| Low green | 100 | chr20:61446962-61447661 | 16 | *COL9A3* | 7.61E-04 | -0.36 | Low green | 100 | chr20:61447035-61447369 | 8 | *COL9A3* | 1.67E-05 | -0.45 |
| Low green | 100 | chr22:31318103-31318546 | 9 | *MORC2-AS1* | 2.71E-02 | 0.77 | Low green | 100 | chr22:31318239-31318444 | 5 | *MORC2-AS1* | 8.01E-04 | 0.55 |
| Low green | 100 | chr22:41763093-41763512 | 8 | *TEF* | 2.16E-04 | -0.25 | Low green | 100 | chr22:41763092-41763512 | 9 | *TEF* | 3.24E-07 | -0.22 |
| Low green | 100 | chr22:42077939-42078723 | 7 | *SNU13* | 9.64E-04 | -0.51 | Low green | 100 | chr22:42078364-42078723 | 5 | *SNU13* | 1.12E-06 | -0.70 |
| Low green | 100 | chr3:194090132-194091167 | 6 | *LRRC15* | 3.49E-06 | -0.88 | Low green | 100 | chr3:194090476-194090730 | 4 | *LRRC15* | 4.09E-08 | -1.10 |
| Low green | 100 | chr5:102898223-102898733 | 4 | *NUDT12* | 6.39E-03 | -1.22 | Low green | 100 | chr5:102898462-102898733 | 3 | *NUDT12* | 1.56E-05 | -1.56 |
| Low green | 100 | chr6:42927504-42928546 | 21 | *GNMT* | 9.07E-04 | -0.38 | Low green | 100 | chr6:42927939-42928144 | 9 | *GNMT* | 1.30E-05 | -0.67 |
| Low green | 100 | chr6:166232637-166233141 | 5 | *LINC00473* | 1.00E-03 | -0.81 | Low green | 100 | chr6:166232959-166233141 | 4 | *LINC00473* | 1.17E-06 | -0.92 |
| Low green | 100 | chr8:22561149-22561238 | 3 | *PEBP4* | 1.71E-02 | -0.26 | Low green | 100 | chr8:22561148-22561238 | 3 | *PEBP4* | 1.56E-05 | -0.26 |
| Low green | 100 | chr8:26047392-26048414 | 7 | *PPP2R2A* | 4.05E-04 | -0.47 | Low green | 100 | chr8:26047779-26048065 | 5 | *PPP2R2A* | 3.89E-07 | -0.61 |
| Total green | 1000 | chr1:29139021-29139522 | 4 | *OPRD1* | 5.62E-03 | 0.09 | Total green | 1000 | chr1:29139020-29139121 | 3 | *OPRD1* | 3.29E-05 | -0.03 |
| Total green | 1000 | chr1:46712613-46713440 | 8 | *RAD54L* | 5.05E-03 | -0.07 | Total green | 1000 | chr1:46712831-46712916 | 2 | *RAD54L* | 2.00E-02 | -0.06 |
| Total green | 1000 | chr11:60414689-60414918 | 3 | *LINC00301* | 1.79E-02 | 1.03 | Total green | 1000 | chr11:60414688-60414918 | 3 | *LINC00301* | 1.97E-05 | 1.03 |
| Total green | 1000 | chr12:9217079-9217907 | 11 | *LINC00612* | 1.45E-05 | 1.50 | Total green | 1000 | chr12:9217509-9217907 | 9 | *LINC00612* | 6.28E-08 | 1.73 |
| Total green | 1000 | chr13:28194706-28194884 | 6 | *LNX2* | 5.80E-03 | -0.06 | Total green | 1000 | chr13:28194705-28194884 | 7 | *LNX2* | 2.46E-05 | 0.03 |
| Total green | 1000 | chr13:41240516-41240761 | 2 | *FOXO1* | 1.04E-02 | -0.04 | Total green | 1000 | chr13:41240515-41240761 | 3 | *FOXO1* | 1.97E-05 | -0.04 |
| Total green | 1000 | chr13:47470793-47471562 | 9 | *HTR2A* | 5.05E-03 | -0.27 | Total green | 1000 | chr13:47470988-47471197 | 5 | *HTR2A* | 1.18E-05 | -0.36 |
| Total green | 1000 | chr13:112838528-112838970 | 4 | *SOX1* | 1.88E-02 | 0.46 | Total green | 1000 | chr13:112838527-112838611 | 3 | *SOX1* | 1.49E-05 | 0.62 |
| Total green | 1000 | chr14:93698870-93699254 | 4 | *UBR7* | 2.18E-02 | 0.35 | Total green | 1000 | chr14:93698869-93698944 | 3 | *UBR7* | 2.70E-04 | 0.41 |
| Total green | 1000 | chr16:34777682-34778087 | 3 | *TP53TG3HP* | 2.22E-03 | 0.84 | Total green | 1000 | chr16:34777880-34778087 | 2 | *TP53TG3HP* | 1.49E-05 | 1.06 |
| Total green | 1000 | chr17:78747227-78748494 | 5 | *RPTOR* | 1.57E-03 | 0.71 | Total green | 1000 | chr17:78747933-78748077 | 3 | *RPTOR* | 1.18E-05 | 0.74 |
| Total green | 1000 | chr19:11266334-11267029 | 7 | *SPC24* | 2.18E-02 | 0.02 | Total green | 1000 | chr19:11266492-11266500 | 2 | *SPC24* | 6.25E-03 | -0.04 |
| Total green | 1000 | chr19:58878570-58879059 | 4 | *ZNF837* | 5.95E-03 | 1.12 | Total green | 1000 | chr19:58879021-58879059 | 3 | *ZNF837* | 1.64E-05 | 1.41 |
| Total green | 1000 | chr22:46685472-46685728 | 4 | *TTC38* | 1.67E-02 | -0.41 | Total green | 1000 | chr22:46685471-46685728 | 5 | *TTC38* | 2.34E-05 | -0.31 |
| Total green | 1000 | chr3:11267020-11267098 | 3 | *HRH1* | 9.11E-03 | 0.80 | Total green | 1000 | chr3:11267019-11267098 | 5 | *HRH1* | 1.49E-05 | 0.54 |
| Total green | 1000 | chr4:77069462-77070069 | 8 | *NUP54* | 5.24E-03 | -0.08 | Total green | 1000 | chr4:77069461-77069719 | 7 | *NUP54* | 1.36E-04 | -0.02 |
| Total green | 1000 | chr4:124318523-124319402 | 3 | *SPRY1* | 1.37E-03 | -0.02 | Total green | 1000 | chr4:124319258-124319402 | 3 | *SPRY1* | 1.49E-05 | 0.00 |
| Total green | 1000 | chr5:110062343-110062837 | 7 | *TMEM232* | 6.29E-03 | -1.42 | Total green | 1000 | chr5:110062342-110062473 | 5 | *TMEM232* | 1.57E-05 | -1.47 |
| Total green | 1000 | chr5:127873106-127873397 | 3 | *FBN2* | 6.83E-03 | -0.07 | Total green | 1000 | chr5:127873105-127873397 | 3 | *FBN2* | 1.64E-05 | -0.07 |
| Total green | 1000 | chr6:31275267-31276187 | 15 | *HLA-DRB5* | 1.62E-04 | 1.15 | Total green | 1000 | chr6:31275550-31275881 | 10 | *HLA-DRB5* | 1.18E-05 | 1.51 |
| Total green | 1000 | chr8:1772264-1772393 | 2 | *ARHGEF10* | 1.14E-02 | -0.12 | Total green | 1000 | chr8:1772263-1772393 | 3 | *ARHGEF10* | 1.64E-05 | 0.05 |
| High green | 1000 | chr1:145095823-145096723 | 11 | *NBPF20* | 1.44E-02 | -0.09 | High green | 1000 | chr1:145096318-145096403 | 4 | *NBPF20* | 2.21E-05 | -0.09 |
| High green | 1000 | chr10:130726406-130726701 | 3 | *MGMT* | 2.92E-02 | 1.43 | High green | 1000 | chr10:130726405-130726701 | 3 | *MGMT* | 2.21E-05 | 1.43 |
| High green | 1000 | chr11:126173246-126174041 | 11 | *DCPS* | 3.94E-03 | 0.36 | High green | 1000 | chr11:126173515-126173568 | 3 | *DCPS* | 4.96E-04 | 0.81 |
| High green | 1000 | chr12:9217079-9217907 | 8 | *LINC00612* | 9.52E-05 | 1.60 | High green | 1000 | chr12:9217528-9217907 | 7 | *LINC00612* | 1.16E-07 | 1.81 |
| High green | 1000 | chr12:54763081-54763433 | 4 | *ZNF385A* | 2.28E-02 | 0.61 | High green | 1000 | chr12:54763210-54763387 | 2 | *ZNF385A* | 3.66E-04 | 0.85 |
| High green | 1000 | chr13:45694580-45694882 | 7 | *GTF2F2* | 3.54E-03 | -0.02 | High green | 1000 | chr13:45694579-45694882 | 7 | *GTF2F2* | 1.80E-05 | -0.02 |
| High green | 1000 | chr13:112838152-112838970 | 5 | *SOX1* | 7.82E-03 | 0.40 | High green | 1000 | chr13:112838527-112838611 | 3 | *SOX1* | 5.80E-06 | 0.67 |
| High green | 1000 | chr14:92959873-92959939 | 3 | *SLC24A4* | 1.02E-02 | 0.36 | High green | 1000 | chr14:92959872-92959939 | 3 | *SLC24A4* | 1.62E-05 | 0.36 |
| High green | 1000 | chr17:78865087-78865755 | 7 | *RPTOR* | 4.32E-03 | 0.83 | High green | 1000 | chr17:78865262-78865662 | 5 | *RPTOR* | 1.80E-05 | 0.99 |
| High green | 1000 | chr19:58861502-58862398 | 6 | *A1BG-AS1* | 1.22E-04 | 1.23 | High green | 1000 | chr19:58862088-58862398 | 5 | *A1BG-AS1* | 1.89E-05 | 0.49 |
| High green | 1000 | chr2:47150-47889 | 7 | *FAM110C* | 4.02E-04 | 0.51 | High green | 1000 | chr2:47656-47889 | 3 | *FAM110C* | 2.38E-05 | 0.74 |
| High green | 1000 | chr2:43327937-43328536 | 4 | *ZFP36L2* | 5.28E-03 | 0.95 | High green | 1000 | chr2:43327936-43328122 | 4 | *ZFP36L2* | 1.80E-05 | 0.72 |
| High green | 1000 | chr2:65593761-65594478 | 5 | *SPRED2* | 3.65E-03 | 0.93 | High green | 1000 | chr2:65593760-65594021 | 4 | *SPRED2* | 5.80E-06 | 1.13 |
| High green | 1000 | chr2:129659018-129659946 | 6 | *HS6ST1* | 5.20E-03 | 0.88 | High green | 1000 | chr2:129659419-129659682 | 3 | *HS6ST1* | 7.62E-05 | 0.97 |
| High green | 1000 | chr3:47952509-47952841 | 3 | *MAP4* | 5.06E-03 | 0.29 | High green | 1000 | chr3:47952610-47952841 | 2 | *MAP4* | 1.80E-05 | 0.37 |
| High green | 1000 | chr4:81117647-81119473 | 12 | *PRDM8* | 9.52E-05 | -1.89 | High green | 1000 | chr4:81119177-81119473 | 5 | *PRDM8* | 4.89E-06 | -1.86 |
| High green | 1000 | chr5:42924215-42924552 | 3 | *LOC648987* | 8.44E-03 | 1.21 | High green | 1000 | chr5:42924366-42924552 | 2 | *LOC648987* | 1.89E-05 | 1.43 |
| High green | 1000 | chr6:26017619-26018602 | 9 | *H1-1* | 2.11E-03 | 0.36 | High green | 1000 | chr6:26018002-26018185 | 5 | *H1-1* | 5.80E-06 | 0.51 |
| High green | 1000 | chr6:31148404-31148666 | 5 | *HLA-DRB5* | 2.88E-02 | 1.34 | High green | 1000 | chr6:31148403-31148666 | 6 | *HLA-DRB5* | 1.89E-05 | 1.15 |
| High green | 1000 | chr8:95962084-95962463 | 6 | *TP53INP1* | 2.16E-02 | -1.04 | High green | 1000 | chr8:95962083-95962463 | 6 | *TP53INP1* | 1.80E-05 | -1.04 |
| High green | 1000 | chr8:144984339-144984599 | 3 | *PLEC* | 1.37E-03 | 0.16 | High green | 1000 | chr8:144984338-144984599 | 3 | *PLEC* | 1.26E-05 | 0.16 |
| Low green | 1000 | chr1:3774827-3775105 | 4 | *DFFB* | 2.05E-02 | 0.60 | Low green | 1000 | chr1:3774826-3775105 | 4 | *DFFB* | 6.08E-06 | 0.60 |
| Low green | 1000 | chr1:92414221-92414520 | 3 | *BRDT* | 2.37E-02 | -1.08 | Low green | 1000 | chr1:92414220-92414520 | 9 | *BRDT* | 9.24E-06 | -0.26 |
| Low green | 1000 | chr10:47134171-47134299 | 2 | *-* | 8.83E-03 | 0.62 | Low green | 1000 | chr10:47134170-47134299 | 6 | *-* | 5.95E-06 | 0.18 |
| Low green | 1000 | chr10:105428385-105428506 | 2 | *SH3PXD2A* | 3.94E-02 | -0.78 | Low green | 1000 | chr10:105428384-105428651 | 4 | *SH3PXD2A* | 9.24E-06 | -0.75 |
| Low green | 1000 | chr11:2889629-2891360 | 33 | *KCNQ1DN* | 4.08E-09 | -0.28 | Low green | 1000 | chr11:2890257-2890725 | 29 | *KCNQ1DN* | 1.38E-08 | -0.28 |
| Low green | 1000 | chr14:91294322-91294412 | 2 | *TTC7B* | 6.70E-03 | -0.43 | Low green | 1000 | chr14:91294321-91294412 | 2 | *TTC7B* | 3.20E-06 | -0.43 |
| Low green | 1000 | chr17:78417789-78418426 | 6 | *ENDOV* | 1.00E-02 | 0.52 | Low green | 1000 | chr17:78417788-78418076 | 5 | *ENDOV* | 5.95E-06 | 0.55 |
| Low green | 1000 | chr19:18979397-18980744 | 5 | *GDF1* | 5.77E-05 | 0.75 | Low green | 1000 | chr19:18980102-18980110 | 2 | *GDF1* | 5.95E-06 | 1.09 |
| Low green | 1000 | chr19:50193021-50194252 | 6 | *ADM5* | 6.00E-07 | 0.54 | Low green | 1000 | chr19:50193856-50194120 | 2 | *ADM5* | 5.95E-06 | 0.72 |
| Low green | 1000 | chr2:27530670-27531360 | 8 | *UCN* | 1.15E-04 | 0.43 | Low green | 1000 | chr2:27531162-27531360 | 7 | *UCN* | 2.46E-06 | 0.38 |
| Low green | 1000 | chr3:183958478-183959853 | 11 | *VWA5B2* | 2.95E-04 | 0.47 | Low green | 1000 | chr3:183958999-183959171 | 7 | *VWA5B2* | 1.35E-06 | 0.58 |

β= mean of coefficients of CpGs included in the region, where coefficients rapresent % increase of DNA methylation per one interquartile range increase of maternal exposure to green space during pregnancy; N= number of CpGs included in the region

## Supplementary Table 5

Numbers of CpGs belonging to HPA axis candidate genes.

|  | **HPA axis genes** | | | | | | |
| --- | --- | --- | --- | --- | --- | --- | --- |
|  | ***CRH*** | ***CRHBP*** | ***FKBP5*** | ***HSD11B2*** | ***NR3C1*** | ***OXTR*** | ***SLC6A4*** |
| *Numbers of CpGs* | 14 | 15 | 32 | 13 | 40 | 16 | 15 |

## Supplementary Table 6

Coefficients and p-values for the association of cg14528046 (located on *PHF17*) and exposure to maternal high green space at 1000m buffer in the main analysis and in the sensitivities adding as covariates i) PM_2.5_, ii) distance to nearest major roads, iii) urbanicity (coded as urban, suburban, and rural), and iv) median neighborhood income.

|  |  |  | **Main analysis** | | **+ PM_2.5_** | | **+ distance from major roads** | | **+ urbanicity** | | **+ income** | |
| --- | --- | --- | --- | --- | --- | --- | --- | --- | --- | --- | --- | --- |
| **CpG** | **Gene name** | **Gene Region** | **coef** | **p-val** | **coef** | **p-val** | **coef** | **p-val** | **coef** | **p-val** | **coef** | **p-val** |
| cg14528046 | *PHF17* | Body | 0.48 | **5.68E-08** | 0.49 | **5.52E-08** | 0.48 | **5.48E-08** | 0.47 | 1.67E-07 | 0.47 | **1.01E-07** |

P-values in bold are genome-wide significant (FDR-adjusted p-values <0.05). coef=% increase of DNA methylation per one interquartile range increase of maternal exposure to green space during pregnancy; p-val= p-value.

## Supplementary Table 7

Coefficients and p-values for the association of cg14528046 (located on *PHF17*) and exposure to maternal high green space at 1000m buffer in the main analysis and in the robustness analyses (using non-trimmed DNA methylation, and in single EWASs in the 450K and EPIC subsets).

|  |  |  | **Main anaysis** | | **Non-trimmed DNA methylation** | | **450K EWAS** | | **EPIC EWAS** | |
| --- | --- | --- | --- | --- | --- | --- | --- | --- | --- | --- |
| **CpG** | **Gene name** | **Gene Region** | **coef** | **p-val** | **coef** | **p-val** | **coef** | **p-val** | **coef** | **p-val** |
| cg14528046 | *PHF17* | Body | 0.48 | **5.68E-08** | 0.39 | 0.12 | 0.50 | 1.81E-04 | 0.46 | 1.41E-04 |

P-values in bold are genome-wide significant (FDR-adjusted p-values <0.05). coef=% increase of DNA methylation per one interquartile range increase of maternal exposure to green space during pregnancy; p-val= p-value.

## Supplementary Table 8

Summary of the results for the differentially methylated region, annotated to *ZNF274*, that was commonly significant below the FDR corrected p-value threshold of 0.05 and the direction of mean methylation of their CpGs was consistent in 450K and EPIC single EWASs, and in the meta-analyses (only results from DMRcate are shown).

|  |  | **Meta-EWASs** | | | **450K** | | | **EPIC** | | |
| --- | --- | --- | --- | --- | --- | --- | --- | --- | --- | --- |
| **Nearest gene** | **Buffer** | **Genomic location** | **FDR from DMRcate** | **β** | **Genomic location** | **FDR from DMRcate** | **β** | **Genomic location** | **FDR from DMRcate** | **β** |
| *ZNF274* | Total green 100 m | chr19:58715251-58716517 | 1.39E-16 | 1.06 | chr19:58715251-58716517 | 7.57E-05 | 1.24 | chr19:58715251-58716517 | 2.37E-06 | 1.01 |

β= mean of coefficients of CpGs included in the region, where coefficients rapresent % increase of DNA methylation per one interquartile range increase of maternal exposure to green space during pregnancy
